# Fostering cell–cell interactions and integrating angiocrine factors to promote the development of salivary microtissues in 3D

**DOI:** 10.1002/btm2.70118

**Published:** 2026-02-18

**Authors:** Mugdha Pol, Apoorva S. Metkari, Stephen M. Frazier, Joshua B. Macugay, Hanyuan Gao, Robert L. Witt, David M. Cognetti, Charles‐Antoine Assenmacher, Xinqiao Jia

**Affiliations:** ^1^ Department of Biological Sciences University of Delaware Newark Delaware USA; ^2^ Department of Materials Science and Engineering University of Delaware Newark Delaware USA; ^3^ Helen F. Graham Cancer Center and Research Institute Christiana Care Newark Delaware USA; ^4^ Department of Otolaryngology Thomas Jefferson University Philadelphia Pennsylvania USA; ^5^ Comparative Pathology Core, School of Veterinary Medicine University of Pennsylvania Philadelphia Pennsylvania USA; ^6^ Department of Biomedical Engineering University of Delaware Newark Delaware USA; ^7^ Delaware Biotechnology Institute Newark Delaware USA

**Keywords:** angiocrine factors, cell–cell interactions, endothelial cells, hydrogels, microtissues, salivary gland stem/progenitor cells

## Abstract

Salivary gland development requires vascular input signals. However, whether endothelial cell‐secreted angiocrine factors have any inductive effects on adult human salivary gland stem/progenitor cells (hS/PCs) is unknown. To advance the engineering of functional salivary glands, we probed the effects of epithelial and endothelial crosstalk on the growth and differentiation of hS/PCs. Culture of hS/PCs in agarose microwells led to the formation of multicellular spheroids with close cell–cell contacts. Compared with the day 3 culture maintained in the hepatocyte media (HEP) typically used for hS/PCs, 7‐day culture in the endothelial cell growth media (EMG2), significantly increased the expression of genes encoding the E‐cadherin (*CDH1*), stem/progenitor markers (*KRT5*, *KRT14*, *MYC*), acinar markers (*AQP5*, *AMY, SLC12A1*), and extracellular matrix proteins (*LAMA1*, *FN1*). Subsequent cultivation of hS/PC spheroids in a hyaluronic acid (HA)‐derived, cell‐adhesive, and proteolytically degradable hydrogel yielded hydrogel‐encapsulated microtissues with complex, multilobulated structures, consisting of fibronectin‐encased lobules connected by F‐actin structures. Addition of a CD31+/vWF+ endothelial cell monolayer on top of the hS/PC‐laden gel construct led to the development of salivary gland microtissues containing differentiated cells expressing key acinar and ductal cell markers. *In vivo* work showed that the cell‐free HA gels implanted in the partially resected rat parotid gland were degraded in 21 days and did not adversely affect the native tissue structure. Collectively, fostering epithelial cell–cell interaction and integrating endothelial cell‐secreted angiocrine signals led to the development of pro‐acinar salivary gland mimetics from adult salivary gland stem/progenitor cells.

## Graphical abstract

Coculturing adult human salivary gland stem/progenitor cells with endothelial cells without direct epithelial‐endothelial cell–cell contacts led to the development of pro‐acinar salivary gland mimetics through paracrine signaling.

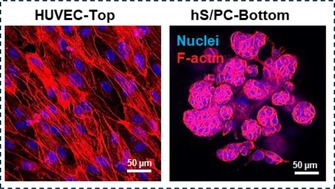

**Translational Impact Statement**

This project seeks to develop an alternative treatment method for xerostomia (or dry mouth), a devastating side effect of radiation therapy that affects 68%–85% of head and neck cancer patients. We aim to recreate a functional salivary gland using patient‐derived progenitor cells and artificial extracellular matrices that recapitulate the native microenvironments of the developing organ. Here, we demonstrate the capacity of the engineered environment with endothelial cell‐secreted factors to promote the development of lobular epithelial structures with differentiated acinar cells.

## 1 INTRODUCTION

Salivary glands, composed of spherical acini connected to tubular conduits in a tree‐like configuration, produce, transport, and secrete saliva to maintain oral homeostasis.^1^ Salivary glands can be damaged by radiation therapy for head and neck cancers, causing persistent dry mouth (or xerostomia) in 68%–85% of patients.^2^ Owing to reduced saliva production and altered saliva composition, patients' quality of life is severely compromised. Symptoms include oral dryness, difficulty in speech and swallowing, and the development of dental caries and periodontal diseases.^3^ Current clinical solutions to xerostomia aim to protect the salivary gland during radiation treatment, to palliate symptoms using water or oral sialagogues, or to stimulate the remaining salivary gland function using cholinergic muscarinic receptor agonists. None has provided long‐term therapeutic benefits.^4^ Salivary gland tissue engineering offers a promising long‐term regenerative solution to radiation‐induced xerostomia. Implantable salivary gland mimetics developed using a biocompatible hydrogel containing organized salivary gland acini‐like structures^5^ can be implanted in patients post‐radiation to restore function.

We developed engineered salivary glands by encapsulating primary adult human salivary stem/progenitor cells (hS/PCs) in customized, hyaluronic acid (HA)‐based hydrogels and culturing the cellular constructs in a hepatocyte media (HepatoSTIM: HEP) with or without pathway‐specific inhibitors.^6^, ^7^, ^8^, ^9^, ^10^, ^11^, ^12^, ^13^ In the presence of a ROCK inhibitor, singly dispersed hS/PCs grew into pro‐acinar multicellular structures that are correctly polarized.^13^ However, unlike the native gland, the multicellular structures developed in these hydrogels are not densely packed, interconnected, or hierarchically organized in a tree‐like architecture. During the initial 3 days of culture, hS/PCs reside in the synthetic matrix as isolated single cells without intimate cell–cell contacts that are crucial to the maintenance of epithelial cell phenotype and function. On the other hand, self‐assembled multicellular structures recapitulate the architecture and organization of developing tissues with increased cell–cell contacts^14^; depending on the location, the strength and the degree of cell–cell and cell–ECM interactions vary. It is shown that primary progenitor cells show enhanced viability and performance when cultured as spheroids. Compared with single cells, self‐assembled spheroids exhibit gene expression profiles that are more reminiscent of the native tissues.^15^

Successful engineering of functional tissues requires adaptation and reactivation of key developmental programs.^16^, ^17^, ^18^ During salivary gland formation in the embryo, intimate crosstalk between the differentiating gland epithelial cells and the surrounding vasculature helps form the hierarchical structure of the mature gland.^19^ Tissue‐specific vascular niches, comprised of endothelial cells and pericyte support cells of mesenchymal origin, deploy sets of angiocrine factors to guide organ development. Specifically, endothelial cells promote expansion of progenitor cells stained positive for KIT, a receptor tyrosine kinase, and suppress premature ductal differentiation.^20^ Signaling involving vascular endothelial growth factor (VEGF) and its receptor (VEGFR2) is required for salivary gland epithelial patterning.^20^, ^21^ Importantly, early organ patterning does not require a fully functional, perfusable blood vessel; primitive vasculature is sufficient to guide tissue morphogenesis.^21^, ^22^, ^23^, ^24^ Endothelial cells secrete angiocrine factors, such as growth factors, adhesion molecules, and chemokines, that affect other cell types in an organ and promote regeneration. The secretion profile of these factors is tissue‐specific.^25^, ^26^ These secreted factors assist in maintaining stem and progenitor cells in a quiescent state during homeostasis and can promote regeneration following injury, in a tissue‐specific manner.^27^ Angiocrine factors are also known to regulate the proliferation, polarity, and differentiation of epithelial cells.^28^, ^29^, ^30^, ^31^ In adult tissues, epithelium‐associated microvessels not only play an important role in the maintenance of tissue homeostasis but also protect the salivary gland from radiation‐induced tissue damage.^32^

Understanding epithelial‐endothelial crosstalk without direct heterotypic cell–cell contacts is crucial for the development of a viable artificial salivary gland. Herein, we investigate the effects of vascular cues on hS/PC phenotypes. Using human umbilical cord vascular endothelial cells (HUVECs) and hS/PCs, we first identify media and hydrogel conditions appropriate for simultaneously maintaining both cell types. Next, hS/PCs were cultured under scaffold‐free conditions in agarose microwells under various media conditions and culture times to develop mature self‐assembled epithelial spheroids. These pre‐assembled hS/PC spheroids were then encapsulated in a cell‐adhesive, protease‐degradable HA hydrogel, with or without an adherent endothelial cell monolayer. We discovered that culturing pre‐assembled hS/PC spheroids in the HA gel resulted in the development of lobulated and interconnected epithelial structures with fibronectin deposition around individual lobules. Under the tissue mimetic coculture conditions, the endothelial cells positively promote the development of pro‐acinar and pro‐ductal progenitors. Conversely, the epithelial cells also reinforce the function of the endothelial cells. As the first step towards *in vivo* testing of the engineered gland, we evaluated the biocompatibility of the hydrogel carrier in the partially resected parotid gland in rats. This study demonstrates the importance of epithelial cell–cell adhesion, matrix conditions, and soluble angiocrine factors in developing bioengineered salivary glands.

## 2 EXPERIMENTAL METHODS

Detailed experimental methods, including cell isolation/maintenance, 3D cultures, gel implantation, and molecular, cellular, and tissue analyses, are provided in Supplementary Information S1. Primer and antibody information can be found in Tables S1 and S2.

## 3 RESULTS

### 3.1 EGM2 is suitable for the maintenance of hS/PCs and HUVECs

**FIGURE 1:**
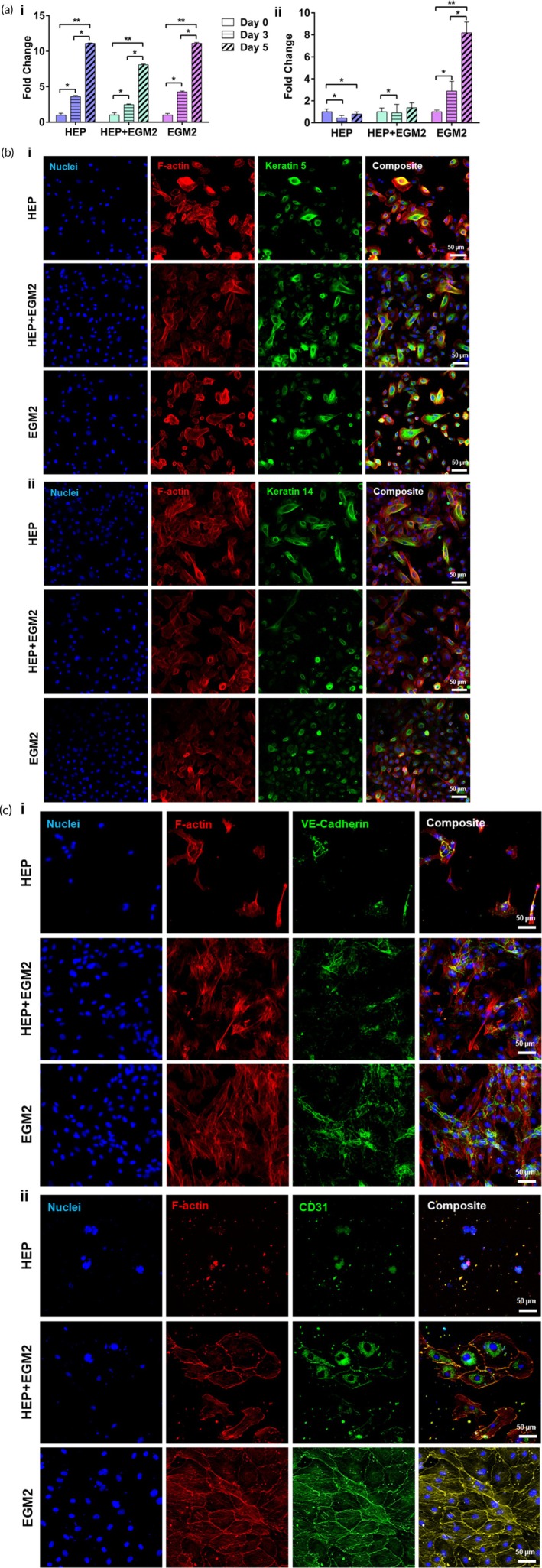
Media screening for 2D maintenance of hS/PCs and HUVECs. (a) Proliferation of hS/PCs (i) and HUVECs (ii) under different media conditions (HEP: 100% HepatoSTIM, HEP + EGM2: 50/50 (v/v) mixture of HepatoSTIM and EGM2, EGM2: 100% EGM2). Data is normalized to the initial number of cells seeded on day 0. Error bars represent SEM, *n* = 3. *p < 0.05, **p < 0.01, one way ANOVA post Hoc Tukey test. (b) Representative confocal images of hS/PCs immunofluorescently stained for keratin 5 (K5, i) and keratin 14 (K14, ii) after 5 days of culture in different media. K5/K14: Green, nuclei: Blue, F‐Actin: Red. Scale bar: 50 μm. (C) Representative confocal images of HUVECs immunofluorescently stained for VE‐cadherin (i) and CD31 (ii) after 5 days of culture in different media. VE‐cadherin/CD31: Green, nuclei: Blue, F‐Actin: Red. Scale bar: 50 μm.

To enable straightforward coculture of the epithelial and the endothelial cells, we first analyzed the behavior of both cell types in HEP, EGM2, and a 50/50 mixture of HEP and EGM2. While hS/PCs proliferated readily in all three types of media (Figures 1ai, S1, and S2), robust and significant cell proliferation of HUVECs was only observed in EGM2 media (Figures 1aii, S3, and S4). hS/PCs maintained in all three types of media consistently expressed the salivary gland stem/progenitor markers keratin 5 (K5, Figure 1bi) and 14 (K14, Figure 1bii). However, for HUVECs, robust expression of endothelial cell markers, vascular endothelial cadherin (VE‐cadherin), an important transmembrane protein that maintains endothelial integrity and regulates vascular permeability,^33^ and platelet endothelial cell adhesion molecule‐1 (PECAM‐1 or CD31), an essential adhesion molecule in endothelial cells, vital for maintaining vascular integrity and promoting angiogenesis,^34^ was only observed in pure EGM2 media (Figure 1ci,ii). The staining for CD31 (Figure 1cii) was sparse and spotty in HEP‐grown HUVECs. While CD31 appeared at the cell–cell junction for EGM2 cultures, the signal was mostly intracellular for cells maintained in the mixed media, indicative of phenotypic changes. Overall, although hS/PCs can tolerate all three media types, dilution of the EGM2 media with HEP compromises endothelial cell function.

### 3.2 EGM2 media promotes the assembly and maturation of hS/PC aggregates

**FIGURE 2:**
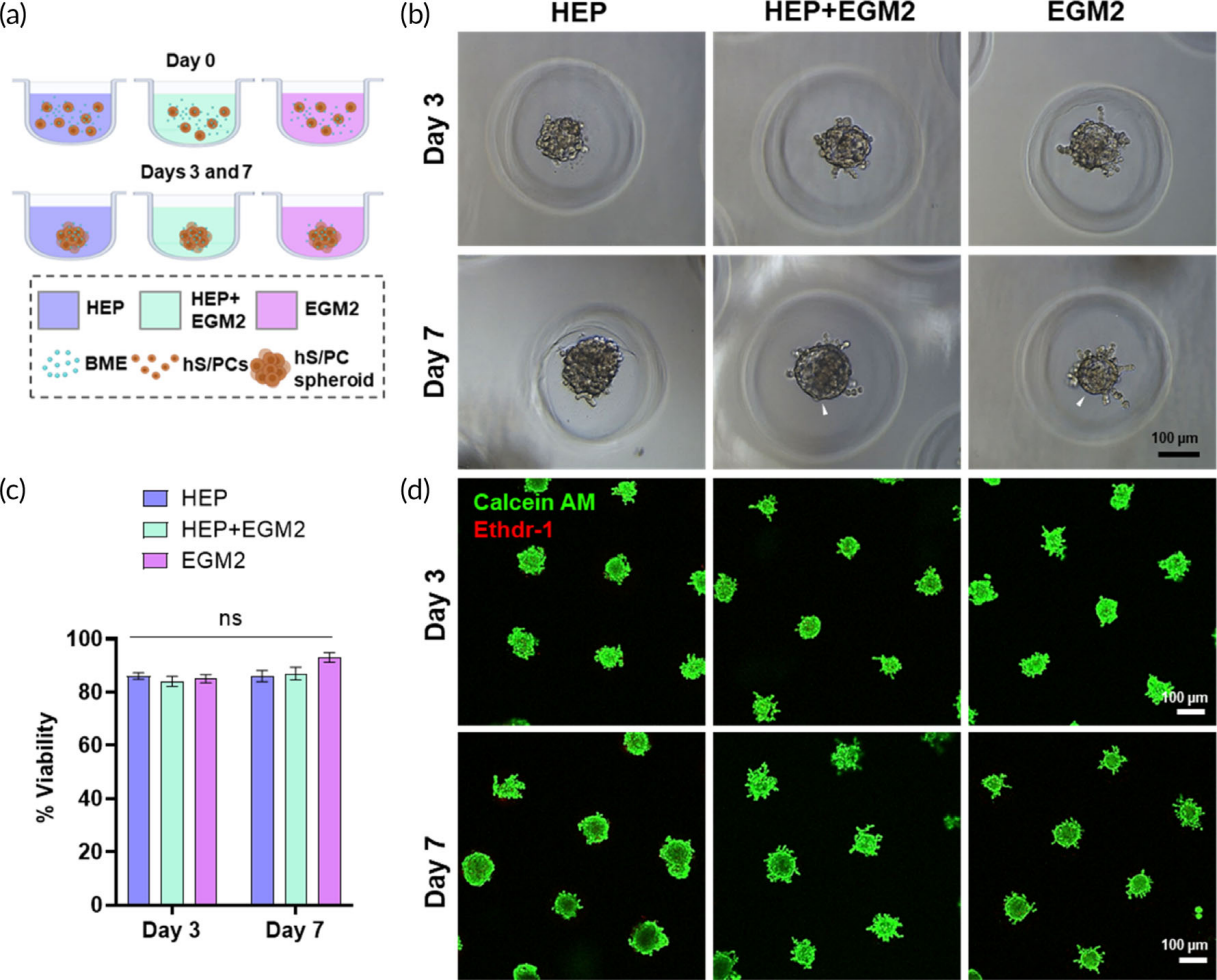
Assembly of hS/PC spheroids in agarose microwells under different media conditions. (a) Schematic depiction of spheroid formation and maturation in various media on days 3 and 7. (b) Bright field images of hS/PC spheroids developed in the agarose microwells. White arrowheads point to the smooth border of the spheroids. Scale bar = 100 μm. (c) Percent viable cells in hS/PC spheroids as a function of culture time and media composition. ns, non‐significant. (d) Representative confocal images of microwell‐grown hS/PC spheroids stained with calcein AM (green‐live cells) and ethidium homodimer‐1 (red‐dead cells). Scale bar = 100 μm.

Spherical and irregularly shaped hS/PC aggregates ranging from 90 to 120 μm were produced in non‐adhesive agarose microwells after 3 and 7 days of culture (Figure 2a), irrespective of the media composition. Strings of several loosely associated cells can be seen radiating from the main body of the cell aggregate (Figure 2b,d). By day 7, structures formed in EGM2‐containing media (EGM2 and EGM2/HEP mixture) exhibit a defined smooth border (white arrow, Figure 2b). Live/Dead staining shows that cells remained viable throughout the culture period, independent of media composition. On average, 84% and 87% of the cells were viable on day 3 and day 7, respectively (Figure 2c,d). Although not statistically significant, day 7 aggregates developed in EGM2 exhibited a higher viability than those formed under other conditions.

**FIGURE 3:**
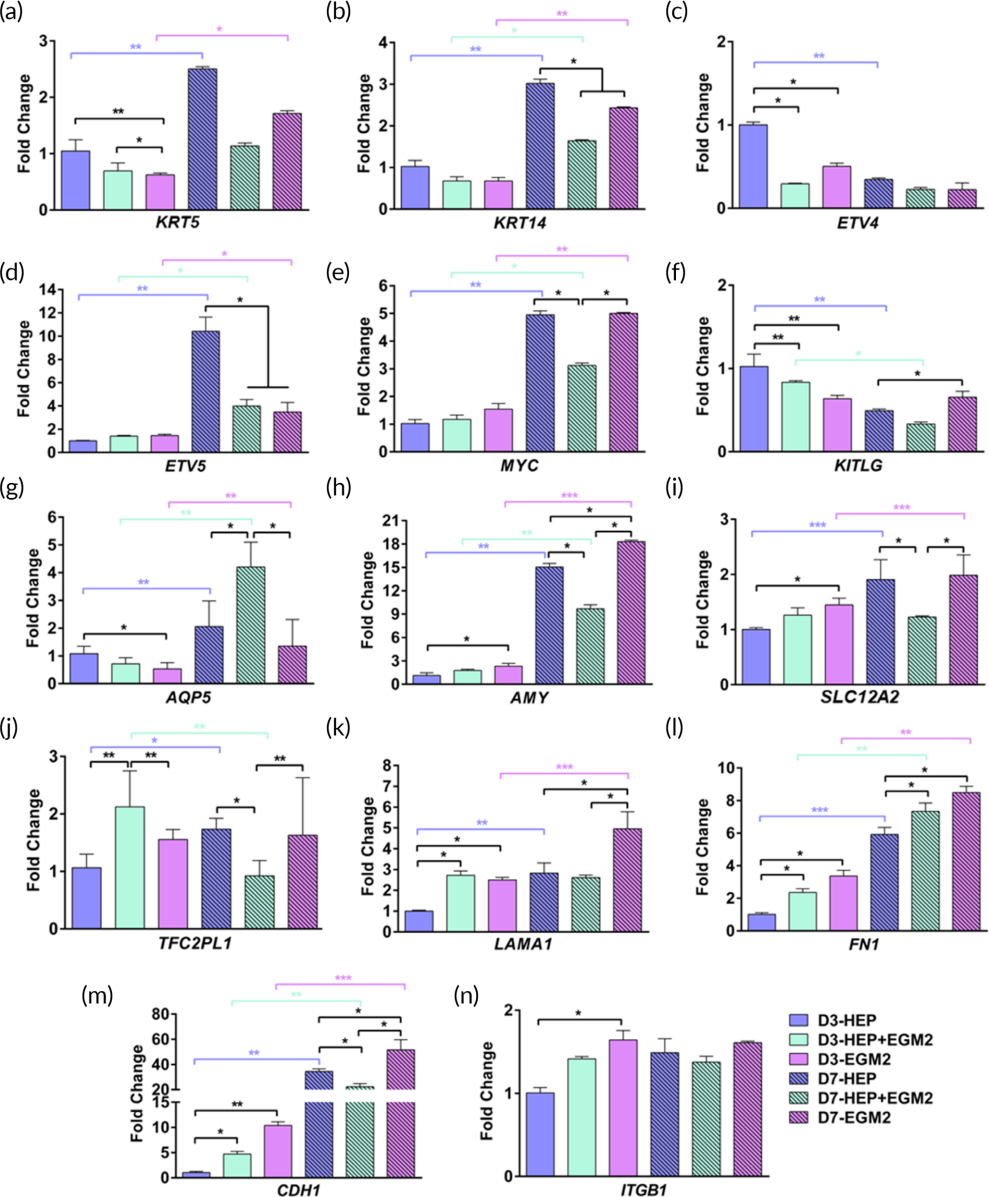
The qPCR analyses of the expression of stem/progenitor markers (*KRT5* – a, *KRT14* – b, *ETV4* – c, *ETV5* – d, *MYC* – e, and *KITLG* – f), differentiation markers (*AQP5* – g, *AMY* – h, *SLC12A2* – i, *TFCP2L1* – j), ECM proteins (*LAMA1* – k, *FN1* – l), cell–cell adhesion protein (*CDH1* – m), and cell‐ECM adhesion protein (*ITGB1* – n) by hS/PC spheroids developed in agarose microwells in various media. Data is normalized to HEP media condition on day 3 of spheroid culture. *n* = 3. Error bars represent SEM, and *GAPDH*  was used as a reference gene. *p < 0.05, **p < 0.01, ***p < 0.005, one‐way ANOVA post‐Hoc Tukey test.

At the transcript level on day 3, the expression of *KRT5*, a stem/progenitor marker, was lower in EGM2 cultures than in HEP cultures on both days 3 and 7. Extending the culture to day 7 increased *KRT5* expression (Figure 3a). A similar expression pattern was observed for another stem/progenitor marker, *KRT14* (Figure 3b). *ETV4* and *ETV5* are the transcription factors required for progenitor cell maintenance in the salivary gland. *ETV4* level was highest in HEP cultures on day 3 (Figure 3c). By contrast, *ETV5* expression remained low across different media conditions on day 3 but increased significantly by day 7 (Figure 3d). Day 7 HEP and EGM2 cultures expressed the highest level of *MYC*, yet another stem/progenitor marker. For EGM2 cultures, the expression was upregulated by 3.3‐fold (*p* < 0.01) from day 3 to day 7 (Figure 3e). Finally, the highest expression level for *KITLG*, KIT ligand, also known as stem cell factor, was detected on day 3 in HEP media. By day 7, the expression level across the three media conditions was comparable (Figure 3f).

RT‐qPCR analysis revealed that day 7 HEP/EGM2 cultures expressed the highest level of *AQP5*, an acinar marker, and all media conditions exhibited a significant (*p* < 0.01) increase in *AQP5* expression on day 7 compared with day 3 (Figure 3g). The expression of *AMY*, an α‐amylase‐encoding gene and an acinar marker, was the highest in the day‐7 EGM2 culture. Compared with the respective day 3 media controls, *AMY* expression increased by 15.1 (*p* < 0.01), 5.5 (*p* < 0.01), and 7.9‐fold (*p* < 0.005) in HEP, mixed, and EGM2 media, respectively (Figure 3h). The expression of *SLC12A2*, gene encoding sodium‐potassium‐chloride cotransporter 1 (NKCC1) and an acinar marker, was higher on day 7 than on day 3, and EGM2 induced a similar level of expression as HEP on day 7 (Figure 3i). The HEP/EGM2 cultures expressed the highest and the lowest levels of *TFCP2L1*, a transcription factor in ductal cell specification (especially intercalated ducts), on day 3 and day 7, respectively (Figure 3j).

Next, we analyzed genes encoding ECM proteins (*LAMA1*, laminin α1; *FN1*: fibronectin). From day 3 to day 7, *LAMA1* expression was increased, and the day‐7 EGM2 cultures expressed the highest level of *LAMA1* (Figure 3k). The expression profile for *FN1* followed a similar pattern. Extending the culture time and the addition of EGM2 led to a steady increase in *FN1* expression; again, the highest *FN1* level was detected on day‐7 EGM2 cultures (Figure 3l). Finally, we examined genes encoding proteins involved in cell–cell (*CDH1*, E‐cadherin) and cell–ECM (*ITGB1*, integrin β1) adhesion. The addition of EGM2 led to a significant upregulation of CDH1 on days 3 and 7, with the highest expression detected in EGM2 cultures (Figure 3m). Although the EGM2 cultures expressed a higher level of *ITGB1* (1.6‐fold, *p* < 0.05) than the HEP controls on day 3, *ITGB1* expression was not significantly affected by media composition and culture time (Figure 3n).

**FIGURE 4:**
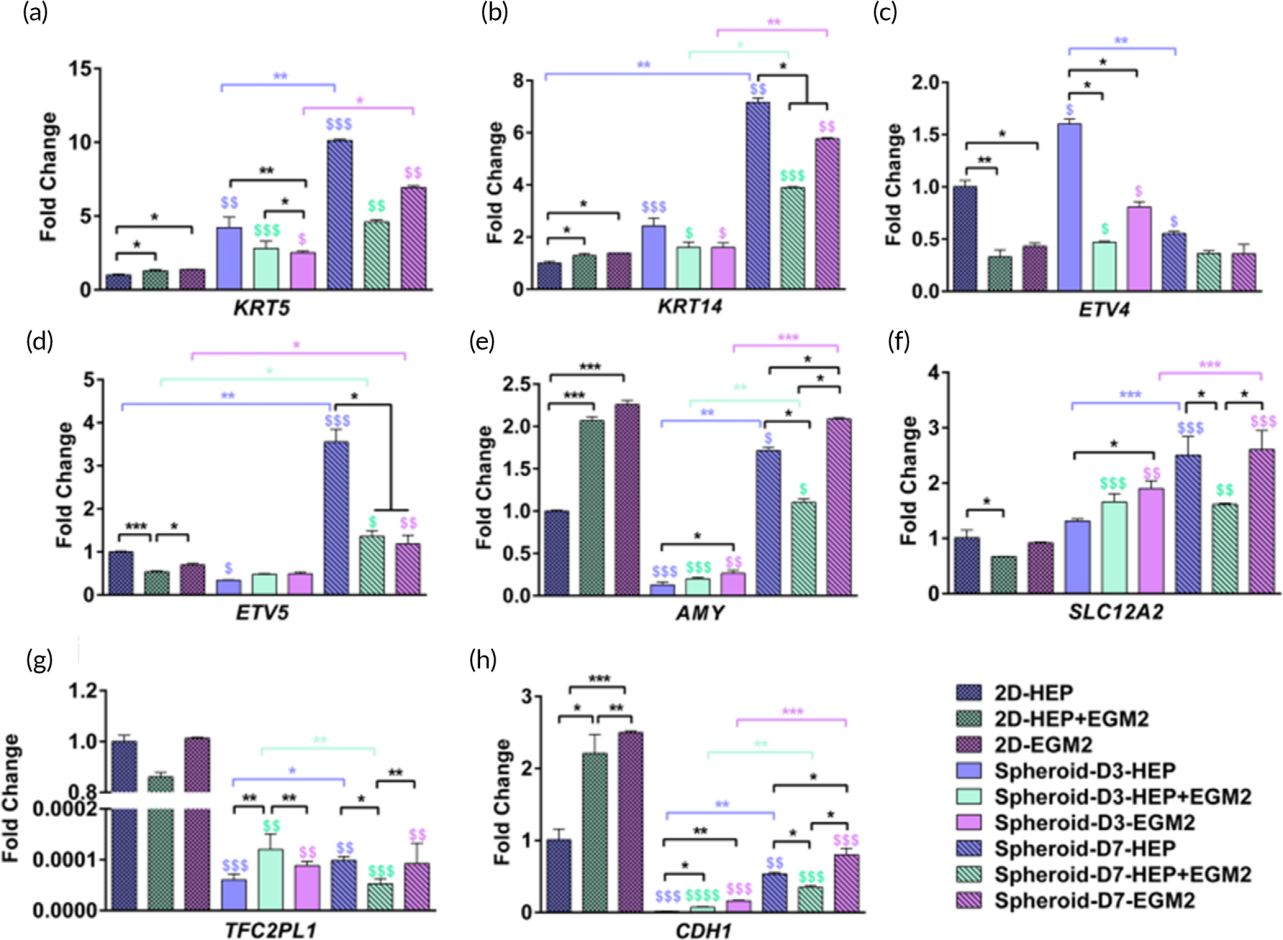
QPCR analyses of the expression of stem/progenitor markers (*KRT5* – a, *KRT14* – b, *ETV4* – c, *ETV5* – d), differentiation markers (*AMY* – e, *SLC12A2* – f, *TFCP2L1* – g), and cell–cell adhesion protein (*CDH1* – h) by hS/PC spheroids developed in agarose microwells and cultured in various media. Results are normalized to HEP media condition for 2D cultures. *n* = 3. Error bars represent SEM, and *GAPDH* was used as a reference gene. Asterisks indicate comparisons between groups indicated by a horizontal bracket, and dollar signs indicate comparisons with the respective 2D cultures. *p < 0.05, **p < 0.01, ***p < 0.005; ^$^ *p* < 0.05, ^$$^ *p* < 0.01, ^$$$^ *p* < 0.005, ^$$$$^ *p* < 0.001; one‐way ANOVA post‐Hoc Tukey test.

Additional experiments were performed to enable comparison of gene expression between spheroid and monolayer cultures (Figure 4). Microwell‐based 3D spheroid cultures on days 3 and 7 significantly increased the expression of *KRT5*, *KRT14*, and *SLC12A2*, but decreased the expression of *TFC2PL1* and *CDH1*. While day 3 spheroids expressed higher levels of *ETV4* (*p* < 0.05), day 7 spheroids had expression levels comparable to those of the respective 2D cultures. Conversely, higher levels of *ETV5* were observed in day 7 spheroids, but not in day 3 spheroids. Compared with 2D cultures, *AMY* expression in hS/PC spheroids was significantly decreased on day 3, then significantly increased by day 7.

At the protein level by immunofluorescence, microwell‐derived hS/PC aggregates were stained positively for K5 and NKCC1 under all media conditions on days 3 and 7 (Figures S5 and S6). Collectively, 7‐day‐old hS/PC aggregates assembled in EGM2 expressed high levels of key stem/progenitor markers (*KRT5*, *KRT14*, *MYC*), acinar markers (*AMY*, *SLC12A2*); maturation of the 3D assemblies is evidenced by the high expression of ECM proteins (*LAMA1 and FN1*) and cell–cell adhesion modules (*CDH1*). Therefore, subsequent experiments were conducted using EGM2‐derived 7‐day‐old hS/PC aggregates.

### 3.3 HA‐based hydrogels support the development of an endothelial cell monolayer

**FIGURE 5:**
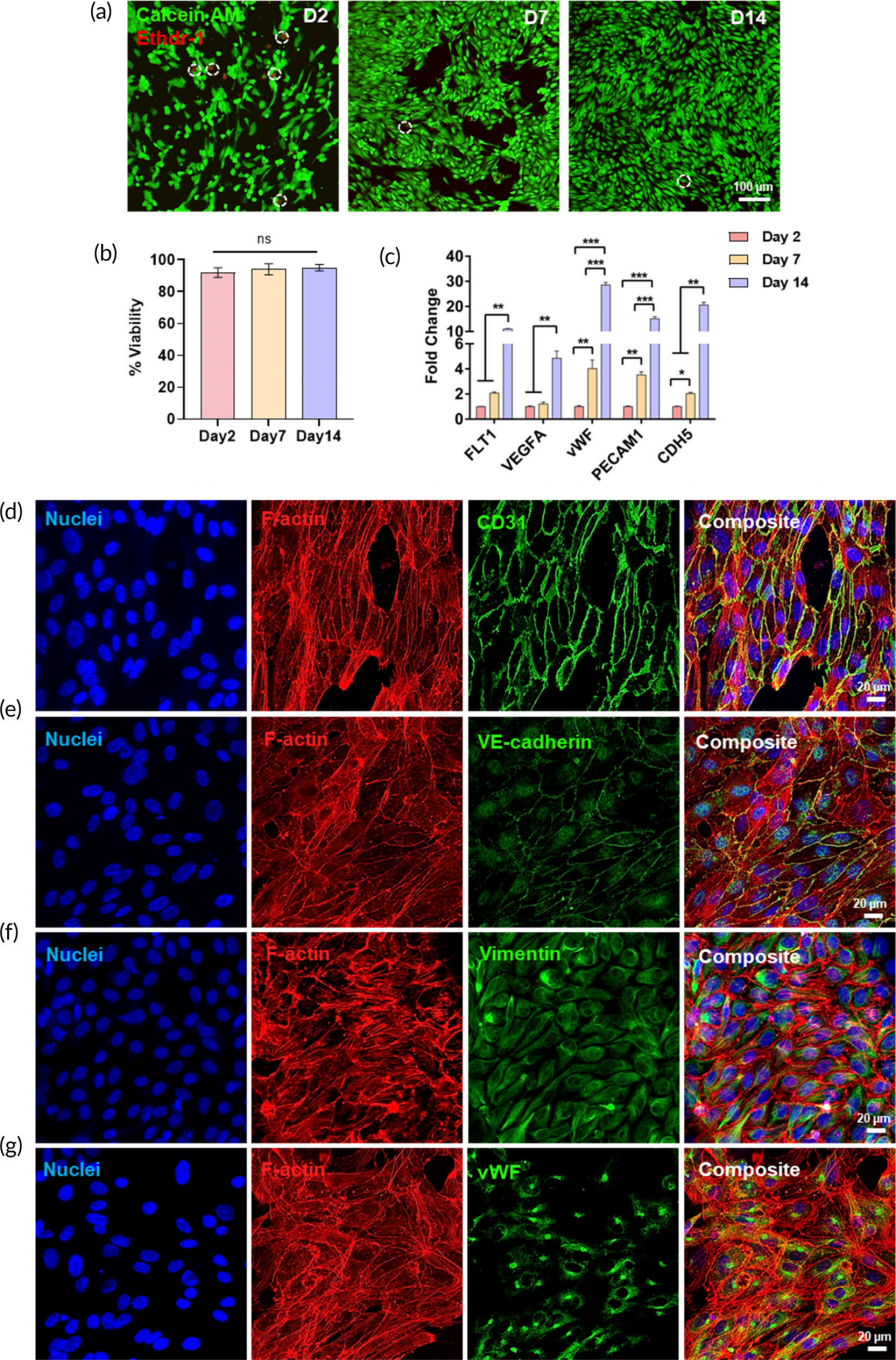
Characterization of HUVEC monoculture maintained on HA hydrogels in EGM2. (a) Representative confocal images of HUVECs stained with calcein AM (green‐live cells) and ethidium homodimer‐1 (red‐dead cells) after 2, 7, and 14 days of culture. White circles indicate dead cells. Scale bar = 100 μm. (b) Percent viable HUVECs as a function of culture time. ns = non‐significant. (c) qPCR analyses of HUVEC‐specific markers across different time points, normalized to day 2. *n* = 3. Error bars represent SEM, *p < 0.05, **p < 0.01, ***p < 0.005, Housekeeping gene: *GAPDH*, one way ANOVA with Post Hoc Tukey test as the reference gene. (d–g) Characterization of HUVEC monocultures by immunofluorescence. CD31, VE‐cadherin, Vimentin and vWF: Green, nuclei: Blue, F‐Actin: Red. Scale bar = 20 μm.

To assess the ability of the synthetic matrix to support endothelial cells, HUVECs suspended in EGM2 were seeded on top of pre‐formed HA hydrogel with RGD. Cells proliferated over time, and by day 14, a confluent monolayer with close cell–cell contacts was established (Figure S7). Cell viability remained high (>92%) over the entire culture period (Figure 5a,b). Upon formation of the cell monolayer, there was a significant increase in the expression of endothelial cell markers. RT‐qPCR experiments (Figure 5c) revealed a 4.9‐fold (*p* < 0.01), and 11.0‐fold (*p* < 0.01) increase in the expression of vascular endothelial growth factor, *VEGFA*, and its receptor *FLT1*, respectively. The expression of the classical endothelial cell marker, von Willebrand Factor (*vWF*), was upregulated by 26.6‐fold (*p* < 0.005). Meanwhile, the expression of cell junction proteins CD31 (*PECAM1*) and VE‐cadherin (*CDH5*) was upregulated by 15.1‐fold (*p* < 0.005) and 20.6‐fold (*p* < 0.01), respectively. At the protein level, HUVECs were stained positive for CD31 and VE‐cadherin, and the fluorescent signals were localized to cell–cell junctions (Figure 5d,e). Bright cytoplasmic stains for intermediate filament, vimentin, were also detected (Figure 5f). When stained for von Willebrand Factor (vWF), the maximum fluorescent signal was concentrated in Weibel‐Palade bodies around the nucleus with a diffuse signal in the cytoplasm (Figure 5g). Collectively, RGD‐containing HA gels support the attachment and growth of endothelial cells.

### 3.4 Pre‐assembled hS/PC spheroids continue to expand in HA gels

**FIGURE 6:**
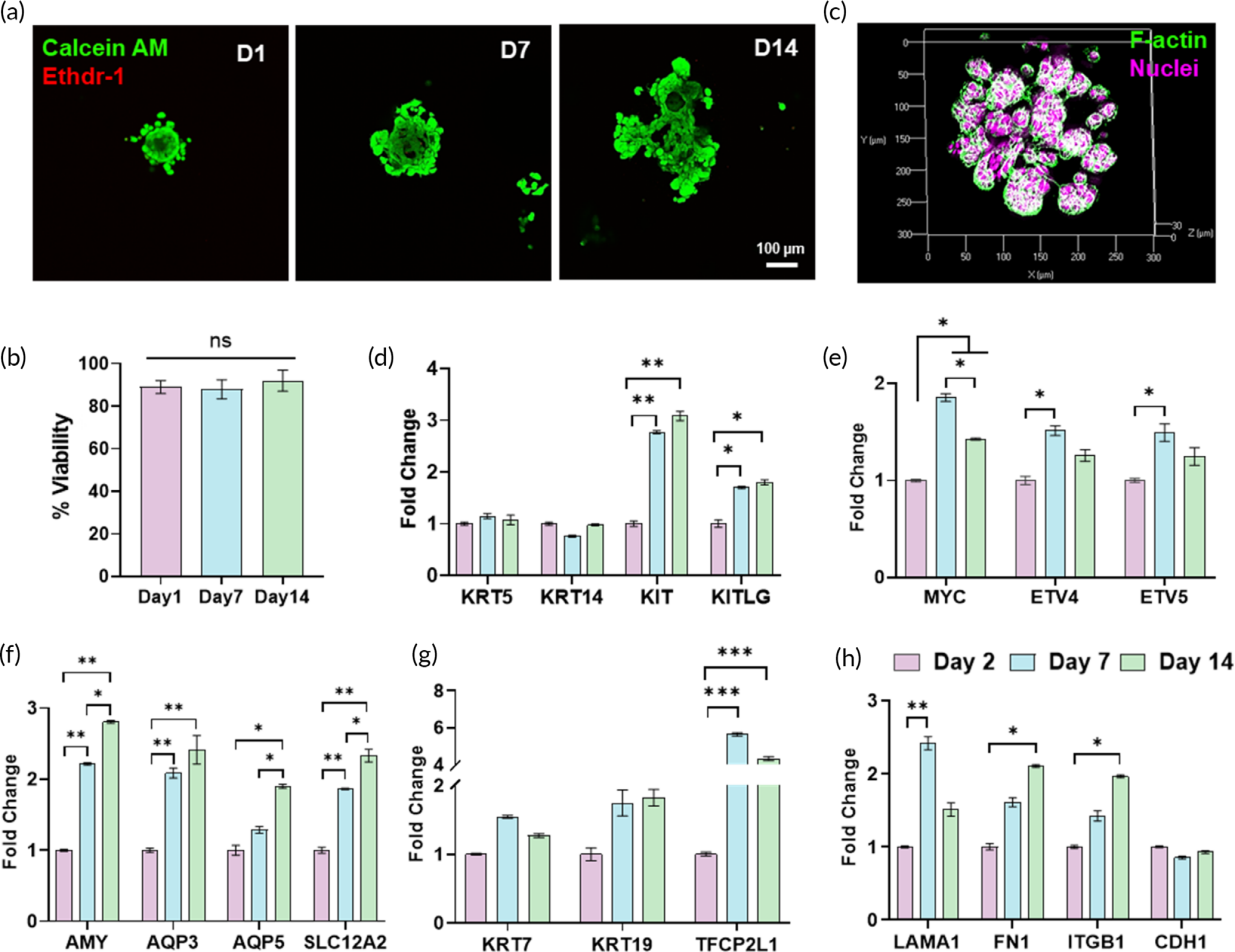
Characterization of hS/PC monocultures maintained in HA gels in EGM2. (a) Representative confocal images of hS/PC spheroids stained with calcein AM (green‐live cells) and ethidium homodimer‐1 (red‐dead cells) after 1, 7, and 14 days of culture. Scale bar = 100 μm. (b) Percent viable cells in hydrogel‐derived hS/PC spheroids as a function of culture time. ns, non‐significant. (c) 3D rendering of a representative confocal image of a complex hS/PC microtissues established in HA gels on day 14. Nuclei: Magenta and F‐Actin: Green. *x*, *y*, *z* dimensions are 300, 300, 30 μm, respectively. (d–h) qPCR analyses of cellular expression of stem/progenitor markers (d, e), differentiation markers (f, g), and ECM, cell–cell, and cell–ECM adhesion proteins (h) across different time points normalized to the day 2 of culture. *n* = 3. Error bars represent SEM, *p < 0.05, **p < 0.01, **p < 0.001, housekeeping gene: *GAPDH*, one way ANOVA with Post Hoc Tukey test.

The pre‐assembled hS/PC spheroids were encapsulated in a soft^35^, ^36^ (*G*′ = 300 Pa) protease‐degradable HA gel containing 2.5 mM RGD peptide, and the resultant 3D epithelial monoculture was maintained in EGM2 media for up to 14 days. Again, cell viability remained high (>88%) throughout the culture period (Figure 6a,b). By day 14, the spherical aggregates had grown into complex lobular structures, where the individual lobules were interconnected with extensive F‐actin structures (Figure 6c). Examination of the 3D hydrogel monoculture by RT‐qPCR revealed that, at the mRNA level, *KRT5* and *KRT14* expressions remained unchanged from day 2 to days 7 and 14 (Figure 6d). Compared with the day 2 level, *KIT* expression was significantly increased on day 7 (2.8‐fold, *p* < 0.01) and day 14 (3.1‐fold, *p* < 0.01). *KITLG* expression followed a similar trend, although the fold increase is more moderate (1.7–1.8‐fold, *p* < 0.05). From day 2 to day 7, cellular expression of *MYC*, *ETV4*, and *ETV5* was increased by 1.9‐fold (*p* < 0.05), 1.5‐fold (*p* < 0.05), and 1.5‐fold (*p* < 0.05), respectively. By day 14, the expression returned to the baseline level on day 2 (Figure 6e).

The 3D hydrogel monoculture of hS/PC spheroids led to a progressive increase in cellular expression of acinar markers over time (Figure 6f). Compared with the day 2 culture, *AMY* expression was increased by 2.2‐fold (*p* < 0.01) and 2.8‐fold (*p* < 0.01) on days 7 and 14, respectively. Similarly, *AQP3* expression was increased by 2.1‐fold (*p* < 0.01) on day 7 and 2.4‐fold (*p* < 0.01) on day 14. Meanwhile, a 1.9‐fold (*p* < 0.05) increase in *AQP5* expression and a 2.3‐fold increase (*p* < 0.01) in *SLC12A2* expression were detected by day 14. While the expressions of early ductal markers *KRT7* and *KRT19* remained unchanged, the expression of a mature ductal marker, *TFCP2L1*, was upregulated by 5.6‐fold (*p* < 0.001) and 4.3‐fold (*p* < 0.001) on days 7 and 14, respectively (Figure 6g). An upregulation of the expression of *LAMA1* and *FN1* was observed on day 7 (2.4‐fold, *p* < 0.01) and day 14 (2.1‐fold, *p* < 0.05), respectively. While a 2.0‐fold increase (*p* < 0.05) in *ITGB1* expression was detected on day 14, *CDH1* expression remained unchanged throughout the culture period (Figure 6h).

**FIGURE 7:**
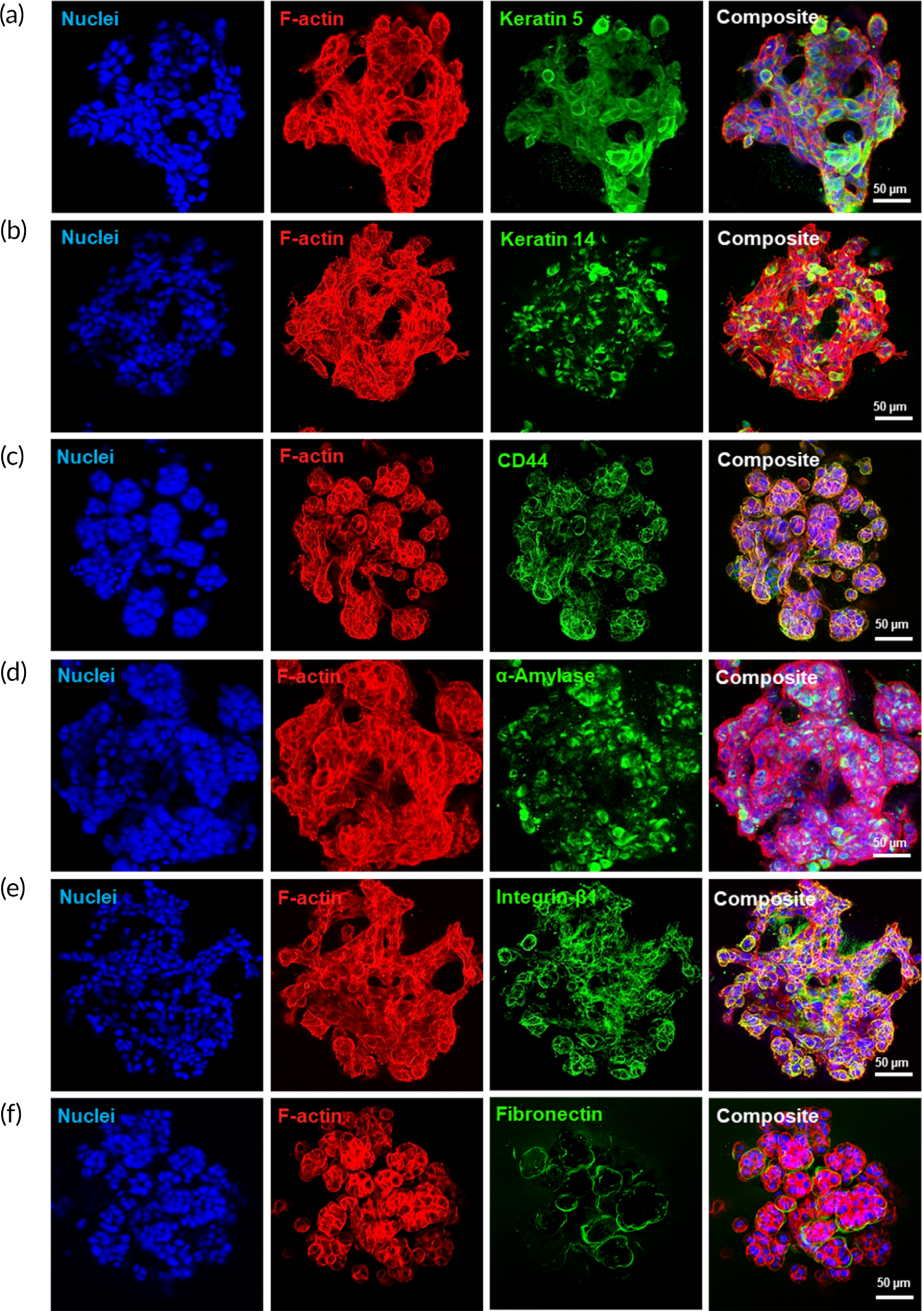
Morphological and phenotypic characterization of hS/PC microtissues established in HA gels. Day 14 microtissues were immunofluorescently stained (green) for keratin 5 (a), keratin 14 (b), CD44 (c), α‐amylase (d), integrin‐β1 (e), and fibronectin (f). Constructs were counterstained for nuclei (blue) and F‐Actin (red). Scale bar = 50 μm.

At the protein level, immunocytochemical analyses showed positive cytoplasmic staining for intermediate filaments K5 (Figure 7a) and K14 (Figure 7b), and positive membrane stains for CD44 (Figure 7c), confirming the maintenance of the stem/progenitor status in the 3D culture. All cells within the lobular structures displayed positive cytoplasmic stains for α‐amylase (Figure 6d). The microtissues were also stained positive for integrin β1 at cell–cell contacts, overlapping with the cortical F‐actin (Figure 7e). Interestingly, the day 14 lobular structures exhibited extracellular deposition of fibronectin around each lobule (Figure 7f). Although some hS/PCs maintained in 2D on tissue culture plates were stained positive for ductal markers K7 and K19 (data not shown), these signals were absent in self‐assembled spheroids. Collectively, after hydrogel encapsulation, the pre‐assembled hS/PC spheroids grew into more complex cellular structures with a multilobulated architecture comprised of pro‐acinar progenitor cells.

### 3.5 HUVECs modulate hS/PC function without direct cell–cell contact

**FIGURE 8:**
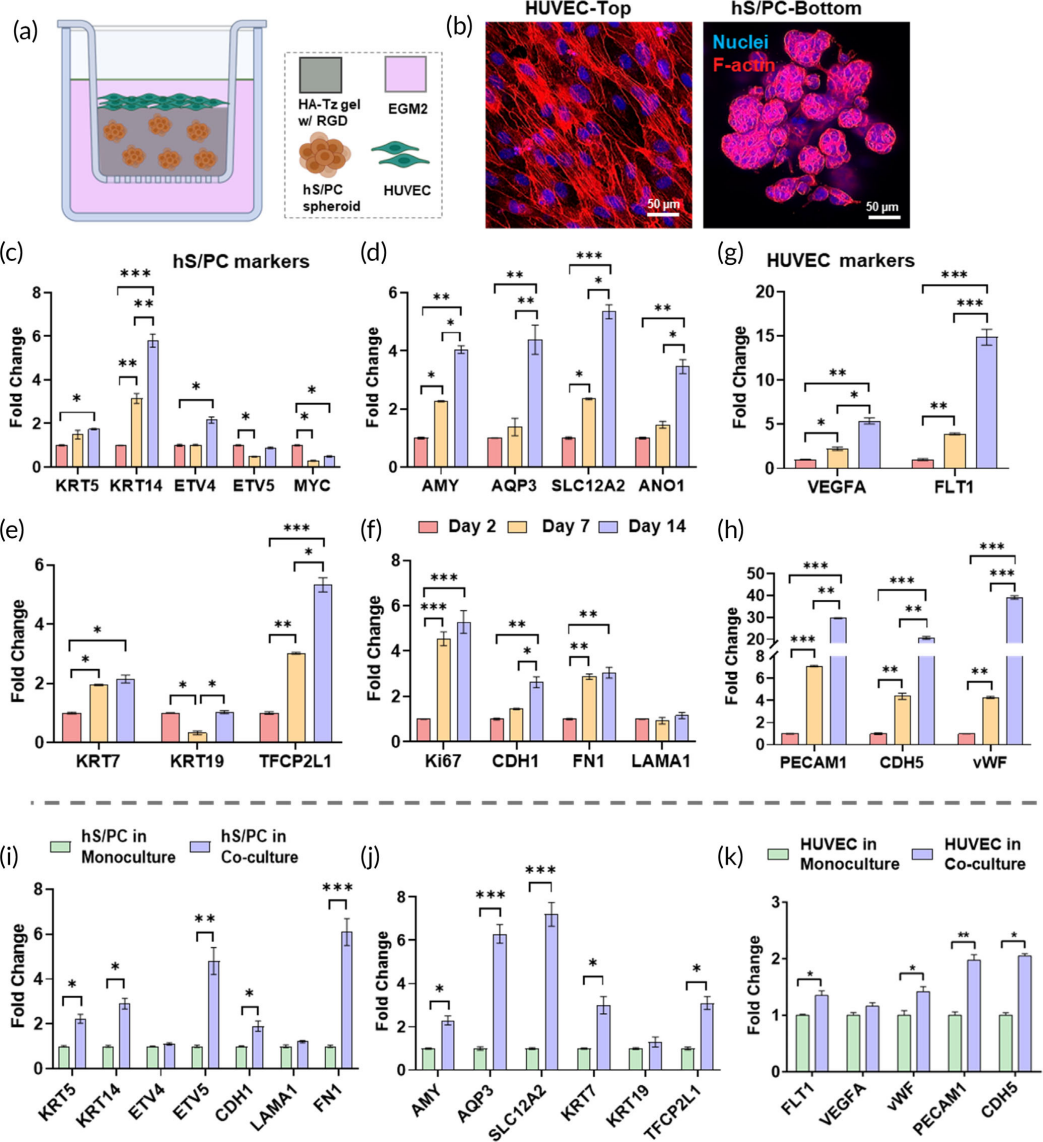
Morphological and phenotypic characterization of cocultured HUVECs and hS/PCs. (a) Schematic representation of the coculture configuration. The culture was maintained in EMG2. (b) Representative confocal images of HUVEC monolayer and hS/PC microtissue established on day 14 on/in HA gels with EGM2. Nuclei: Blue, F‐Actin: Red. Scale bar = 50 μm. (c–f) qPCR analyses of hS/PC expression of epithelial cell‐relevant markers and proteins across different time points, normalized to day 2. (g, h) qPCR analyses of HUVEC expression of endothelial cell‐relevant markers and proteins across different time points, normalized to day 2. (i, j) qPCR analyses of hS/PC expression of epithelial cell‐relevant markers and proteins in epithelial‐endothelial cocultures, normalized to the corresponding epithelial monocultures on day 14. (k) qPCR analyses of hUVEC expression of endothelial cell‐relevant markers and proteins in epithelial‐endothelial cocultures, normalized to the corresponding endothelial monocultures on day 14. For all qPCR analyses, *n* = 3. Error bars represent SEM, *p < 0.05, **p < 0.01, ***p < 0.001, housekeeping gene: *GAPDH*, one‐way ANOVA with Post Hoc Tukey test or Student's *t*‐test.

Epithelial‐endothelial coculture was established by encapsulating pre‐assembled hS/PC spheroids in the bulk HA hydrogel with RGD and seeding HUVECs on top of the epithelial construct (Figure 8a). After 14 days of culture in EGM2, the multicellular epithelial structure and endothelial cell monolayer were morphologically similar to those developed in the respective monocultures (Figure 8b). Endothelial cell infiltration into the epithelial compartment was not observed, and the epithelial cells remained clustered in the hydrogel without extending into the endothelial monolayer. Consequently, the two cell types were readily separated (Figure S8) for RT‐qPCR analysis of their respective phenotypic markers. First, experiments were performed to assess temporal gene expression (Figure 8c–h). Compared with day 2, hS/PCs expressed significantly higher levels of *KRT5* (1.7‐fold, *p* < 0.05), *KRT14* (5.7‐fold, *p* < 0.001), and *ETV4* (2.1‐fold, *p* < 0.05) on day 14. While *ETV5* expression was unchanged, *MYC* expression was decreased (2.0‐fold, *p* < 0.05, Figure 8c). A 14‐day coculture resulted in a significant upregulation of the expression of acinar markers *AMY* (4.1‐fold, *p* < 0.01), *AQP3* (4.4‐fold, *p* < 0.01), *SLC12A2* (5.3‐fold, *p* < 0.001), and *ANO1* (3.4‐fold, *p* < 0.01, Figure 8d). This was accompanied by a 2.1‐fold (*p* < 0.05), and a 5.3‐fold (*p* < 0.001) increase in the expression of ductal markers *KRT7* and *TFCP2L1*, respectively (Figure 8e). Although *LAMA1* expression did not change, a significant upregulation of *Ki67* (5.2‐fold, *p* < 0.001), *CDH1* (2.6‐fold, *p* < 0.01), and *FN1* (3‐fold, *p* < 0.01) was observed (Figure 8f). Importantly, 14‐day coculture in EGM2 resulted in a significant upregulation of all endothelial cell markers examined in HUVECs (Figure 8g,h), including *VEGFA* (5.3‐fold, *p* < 0.01), *FLT1* (14.8‐fold, *p* < 0.001), *PECAM1* (29.6‐fold, *p* < 0.001), *CDH5* (20.6‐fold, *p* < 0.001), and *vWF* (39.1‐fold, *p* < 0.001).

Next, gene expression for the day 14 coculture was compared with the respective monocultures (Figure 8i–k). The addition of endothelial cells resulted in a significant increase in epithelial cell expression of progenitor markers: *KRT5* (2.2‐fold, *p* < 0.05), *KRT14* (2.9‐fold, *p* < 0.05), *ETV5* (4.8‐fold, *p* < 0.01), acinar markers: *AMY* (2.3‐fold, *p* < 0.05), *AQP3* (6.3‐fold, *p* < 0.001), *SLC12A2* (7.2‐fold, *p* < 0.001), and ductal markers: *KRT7* (3.0‐fold, *p* < 0.05), *TFCP2L1* (3.1‐fold, *p* < 0.05). While the expression of *ETV4*, *LAMA1*, *and KRT19* was not affected by the endothelial cells, the expression of *CDH1* and *FN1* was upregulated by 1.9‐fold (*p* < 0.05) and 6.1‐fold (*p* < 0.001), respectively (Figure 8i,j). Inclusion of hS/PCs also had a profound effect on HUVECs (Figure 8k). Specifically, endothelial cells maintained in the coculture expressed a higher level of *FLT1* (1.4‐fold, *p* < 0.05), *vWF* (1.4‐fold, *p* < 0.05), *PECAM1* (2.0‐fold, *p* < 0.01), *and CDH5* (2.0‐fold, *p* < 0.05) than the monoculture. Collectively, juxtaposing the two cell types in a tissue‐mimetic configuration without direct, heterogeneous cell–cell contact enhanced the differentiation and maturation of both cell types.

### 3.6 HA‐based hydrogels are tolerated in partially resected parotid gland

**FIGURE 9:**
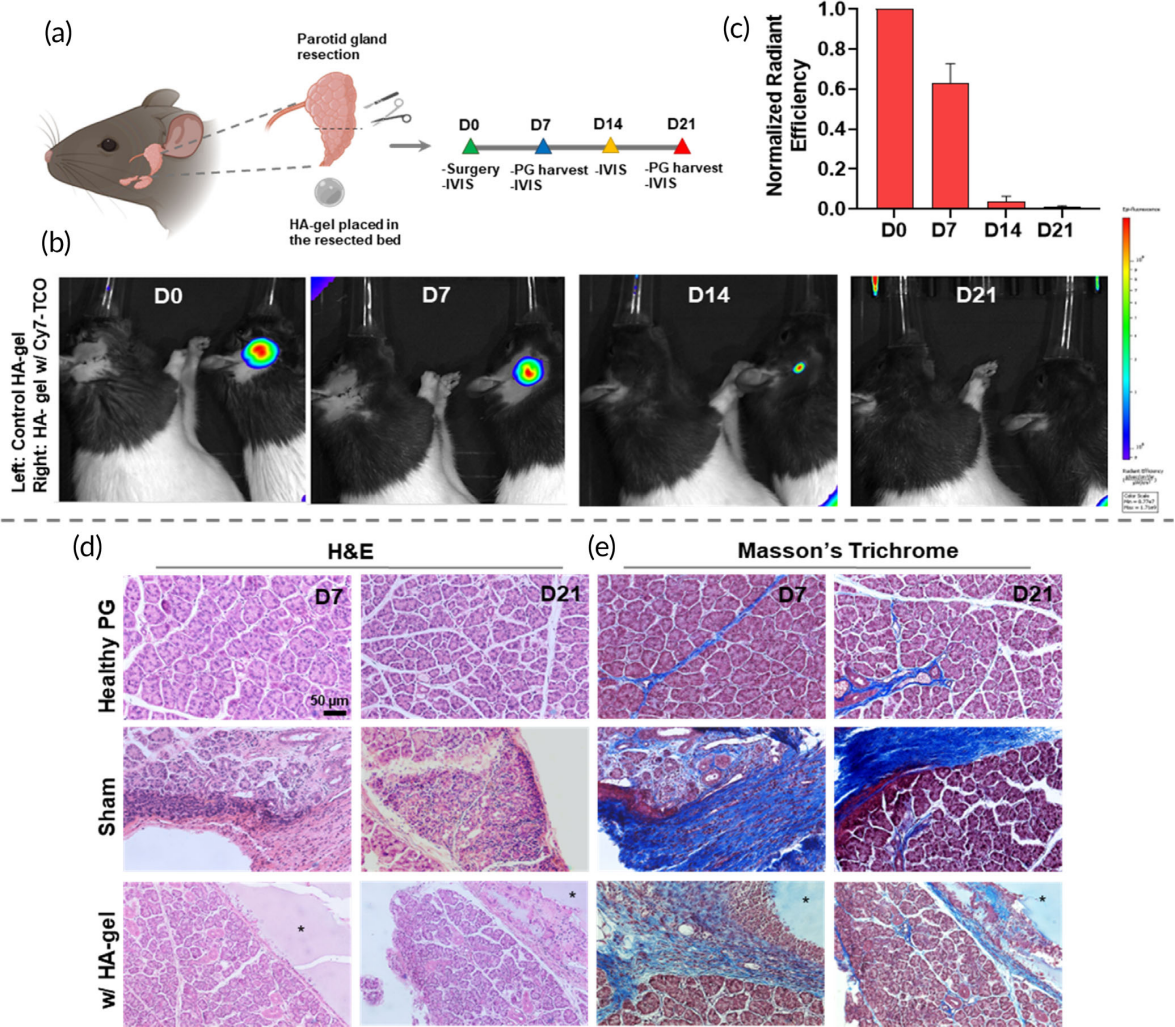
*In vivo* characterization of the HA hydrogel. (a) Schematic representation of parotid gland partial resection and hydrogel implantation. (b, c) Hydrogel *in vivo* residence was assessed by IVIS imaging. The rat on the right side of the image received a Cy7‐labeled gel, whereas the one on the left was treated with a dye‐free gel (b). The part figure c shows normalized radiant efficiency as a function of time. (d, e) Parotid glands were harvested on days 7 and 21 to assess the biocompatibility of cell‐free matrices by H&E (d) and Mason's trichrome (e) staining. Three rats were analyzed for each condition. Healthy PG: No resection and no gel implantation; sham: Resected parotid gland without hydrogel placement; with HA‐gel: Resected parotid gland with HA hydrogel. The asterisk indicates hydrogel fragments. Scale bar = 50 μm.

To simulate acinar cell loss, 1/3 of the rat parotid was resected and replaced with a hydrogel implant (Figure 9a). To enable *in vivo* imaging, hydrogels were labeled with a near‐infrared dye Cy7 before implantation. Figure 9b shows that animals receiving Cy7‐labeled gels displayed a bright fluorescent area ~5 mm in diameter at the implantation site on day 0. No fluorescent signal was detected in the resected parotid in animals receiving dye‐free hydrogels. The radiant efficiency normalized to the day 0 level reduced progressively over time, and by day 21, only small gel fragments remained in the tissue (Figure 9c). Separately, animals receiving hydrogel implantation were sacrificed on days 7 and 21, and the parotid gland was retrieved and processed for H&E and Masson's Trichrome staining. The healthy, untreated parotid gland shows closely packed acini, well‐organized ducts, and minimal stromal infiltration, with an overall lobular architecture (Figure 9d,e). H&E staining for resected glands showed mixed inflammatory cell infiltrates, lobular atrophy, and chronic hemorrhage on day 7 and day 21 after the surgery. Masson's Trichrome evaluation suggested a moderate regional peri‐glandular increase in connective tissue on day 7 after the resection surgery. There was no change in this observation even after 21 days post‐resection surgery.

On day 7, the hydrogel implanted was surrounded by a chronic granulomatous foreign body type inflammatory infiltrate at the edge of the gland, along with a fibrous capsule and mild mixed inflammatory cell infiltrates in the adjacent tissues. Mild multifocal lobular atrophy with ductal dilation and squamous metaplasia was observed. There was a moderate peri‐glandular increase in connective tissue as observed by Masson's Trichrome staining. The fibrous capsule around the glandular defect stained positive for Masson's Trichrome stain. Similar results were seen on day 21 after hydrogel implantation (Figure 9d,e). These results indicate that the HA‐based hydrogel can be biodegraded locally in the parotid gland. The tissue response to the hydrogel implant is mild, and the hydrogel alone did not alleviate the compensatory connective tissue infiltration.

## 4 DISCUSSION

A significant challenge in salivary gland tissue engineering is producing epithelial microtissues with maximized surface area and spatially segregated acini and ducts to enable directional fluid production and transport. We have established protocols for isolating and expanding primary human salivary stem/progenitor cells from the parotid tissues.^8^ These cells can be converted to pro‐acinar/pro‐ductal progenitor cells to recover the secretory function. To produce the engineered gland, hS/PCs were dispersed as single cells in various HA‐based hydrogels.^7^, ^9^, ^10^, ^11^, ^13^ We showed how alteration of the composition, architecture, molecular weight, and protease susceptibility of the hydrogel building blocks affects the clonal expansion of hS/PCs, the maintenance of the progenitor status, and the differentiation into acinar cells.^8^, ^9^, ^11^, ^12^ We further discovered that supplementing the 3D culture with inhibitors that target specific signaling pathways (TGFβ1 and ROCK) profoundly affects hS/PC phenotypes and organizations.^10^, ^13^ Importantly, we discovered that ROCK inhibition led to the establishment of multicellular structures that are correctly polarized.^13^ Occasionally, merged structures with enhanced fibronectin staining at the “cleft” region were seen,^13^ but interconnected lobular structures were not developed.

During embryonic salivary gland development, the tree‐like hierarchical structure is formed through intricate crosstalk between differentiating epithelial cells and surrounding vasculature.^19^ Salivary gland tissue‐specific vascular niches secrete various angiocrine factors that control organ morphogenesis.^19^, ^20^ Early organ patterning does not require a fully functional, perfusable blood vessel network; a primitive vasculature is sufficient to guide organ morphogenesis.^20^, ^22^, ^23^, ^24^ In the mature salivary gland, microvessels closely associated with the epithelium play an important role in protecting the gland from radiation‐induced damage.^37^ To promote hS/PC differentiation and to enable the establishment of the spatially patterned salivary microstructures, self‐assembled hS/PC spheroids were embedded in a cell‐adhesive, protease‐degradable HA hydrogel, and the resultant cellular constructs were cultured in the presence of vascular cues, either from endothelial cell media or the adherent HUVEC monolayer.

Our initial 2D media screening experiments showed that while hS/PCs grew readily in EGM2 media, the HEP media did not support the endothelial cells. This is not surprising because, except for EGF, the HEP media does not contain key angiogenic growth factors, such as fibroblast growth factor‐2 (FGF2), VEGF, insulin‐like growth factor‐1 (IGF1), and ascorbic acid, all indispensable for maintaining endothelial cell functions.^38^, ^39^ These soluble factors did not negatively impact the stem/progenitor status of the epithelial cells. The HA‐based hydrogel, although several orders of magnitude stiffer than gelatin‐coated tissue culture plates, also supported the attachment and the proliferation of HUVEC in EGM2. RGD at a concentration of 2.5 mM is sufficient for cell attachment, eliminating the need for gelatin coating. Upon addition of EGM2, the soluble growth factors in the medium rapidly equilibrated inside and outside of the network with an average pore size of 70–100 nm.^12^, ^40^ Therefore, HUVEC infiltration into the bulk of the HA matrix was not observed.

The hS/PCs form multicellular spheroids in the agarose microwells under all media conditions. Although spheroids were formed on day 3, extending the suspension culture to day 7 led to spheroid compaction, possibly through the reinforcement of cell–cell adhesion (increased *CDH1* expression) and the production of ECM proteins around the cell cluster (increased expression of *LAMA1* and *FN1*). The upregulation of *CDH1* may promote branching and acinar differentiation since blocking of E‐cadherin is shown to inhibit salivary gland branching.^41^, ^42^ Production and accumulation of ECM around the expanding epithelial structure is the first step to building a branched glandular tissue.^43^ In line with our observations, previous work showed that pre‐vascularized dermal spheroids cultured in non‐adhesive microwells for a longer duration exhibited increased expression of fibronectin, laminin, and collagen type I/IV.^44^, ^45^ Alternatively, dental mesenchymal stem cells (MSCs) cultured as preassembled spheroids aided chondrogenic differentiation, and adipose‐derived MSCs spheroids enabled differentiation into hepatocyte‐like cells.^46^, ^47^

Prolonged suspension culture in the EGM2 media also promoted the development of pro‐acinar progenitor cells from hS/PCs, as evidenced by the upregulation of the expression of key stem/progenitor and acinar markers at the transcript level. Here, soluble factors in EGM2 acted as angiocrine signals, contributing to the growth and maturation of the epithelial spheroids. EGF, FGF2, and VEGF have all been shown to be essential in maintaining progenitor populations, regulating epithelial cell proliferation, budding and branching, and stimulating acinar/ductal differentiation.^16^, ^20^, ^48^ Importantly, IGF‐1 injection before radiation treatment has been shown to preserve salivary gland function by activation of endogenous Akt and suppressing apoptosis.^49^, ^50^ In mice, ascorbic acid is shown to increase saliva secretion by upregulation of acetylcholine or β‐adrenergic receptors.^51^

After encapsulation in the HA gel, the self‐assembled hS/PC spheroids continued to expand to establish complex cellular structures with a distinct multi‐lobular morphology. However, individual acini‐like lobules were not connected by ductal structures; instead, extended F‐actin fibers bridged neighboring lobules. The synthetic matrix contains integrin‐binding RGD peptides and MMP‐cleavable crosslinks, promoting cell–ECM interactions. Initially, cells on the outer surface of the spheroids may loosen cell–cell contacts and strengthen cell–ECM adhesion. These cells can multiply and spread into the matrix without being completely detached from the initial aggregate because cell–cell interactions were intact within individual lobules. Under matrix confinement, fibronectin deposition was observed to be spatially restricted to the border of individual lobules. In embryonic salivary gland development, strong cell‐matrix adhesion and weak cell–cell adhesion on the outer side of the spheroid, with increased fibronectin expression in the cleft region, eventually led to budding morphogenesis. These branching structures contain both low and high E‐cadherin cells. High E‐cadherin cells in the core and low E‐cadherin cells on the outer side facilitate integrin β1‐mediated cell‐matrix interaction for successful budding.^16^, ^18^, ^43^ Here, budding morphogenesis is not observed. However, the cell‐permissive matrix continued to reinforce the stem/progenitor status, stimulate the secretion of ECM proteins, and promote the development of pro‐acinar cell populations. Although a significant increase in the expression of *TFCP2L1*, a marker for mature ductal cells, was observed, the 3D culture was stained negative for ductal cell markers K5 and K19 (data not shown).

Adding an adherent HUVEC monolayer further promoted the differentiation and maturation of the bioengineered salivary gland, as evidenced by the significant upregulation of the expression of stem/progenitor markers, acinar markers, ductal markers, ECM protein (FN1), and proteins involved in cell–cell adhesion. Because both the monoculture and the coculture were maintained in EGM2, such an increase can be attributed to paracrine signaling between the epithelial and endothelial cells. The endothelial cell secretome plays a critical role in salivary gland morphogenesis, acting beyond vascular support to influence epithelial branching, progenitor maintenance, and stromal signaling.^20^ Here, angiocrine signals secreted by the cocultured endothelial cells enhanced the bioavailability of factors already in EGM2 (EGF, FGF2, VEGF, IGF). In addition, endothelial cells are known to secrete bone morphogenic proteins (BMPs), such as BMP2 and BMP7, which have been shown to synergize with FGF2 to stimulate proacinar cell differentiation during development.^52^ Endothelial secretomes are also enriched with Wnt and Notch pathway modulators and IGF‐binding proteins (IGFBP2, 3) important in progenitor maintenance and epithelial cell patterning.^20^ Finally, endothelial cells can help remodel the HA network by secreting various MMPs and ECM proteins, thereby rendering the extracellular environment more permissive for the expansion and outgrowth of hS/PC lobules.^53^ During early salivary gland development, the endothelial cell secreted morphogens are spatially and temporally presented. Here, the HUVEC‐secreted modulators are homogeneously distributed in the HA gels. Consequently, the progenitors, ductal (if any), and acinar cells are not spatially compartmentalized. Interestingly, hS/PCs also reciprocally enhanced the function of HUVECs, as evidenced by the upregulation of the expression of classical endothelial cell markers (*FLT1*, *vWF*, *PECAM1*, *and CDH5*).

To establish a hydrogel‐based cellular implant to treat xerostomia, we first assessed the biocompatibility of the cell‐free hydrogel carrier in the resected parotid bed in rats. Owing to the susceptibility of the hydrogel building blocks to enzymatic cleavage (HA by hyaluronidase and SMR‐bisNb by MMPs),^54^ only a small amount of gel fragments remained 21 days post‐implantation. In agreement with literature reports,^55^ the resected gland showed the presence of mixed inflammatory cell infiltrates, lobular atrophy, and typical wound healing responses. With the addition of HA gel, a thin fibrous capsule was seen around the implant, and there was a moderate peri‐glandular increase in connective tissue. However, the hydrogel implant did not improve nor exacerbate the fibrosis. Because the hydrogel was prepared in EGM2, it is reasonable to assume that, as the hydrogels degrade in vivo, soluble factors present in EGM2, which are proangiogenic and antifibrotic,^56^ are released locally. However, our results show that these factors with the specified concentrations in EGM2 are insufficient to mitigate fibrosis, which, according to O'Keefe, persists for up to 56 days after resection surgery.^57^

## 5 CONCLUSION

Although it is well‐accepted that salivary gland development requires input from the surrounding vasculature, no prior studies have examined the effects of endothelial cell‐secreted angiocrine factors on adult human salivary gland stem/progenitor cells. Towards the goal of establishing functional salivary gland microtissues, we conducted 3D coculture studies in a tissue mimetic configuration using hS/PCs and HUVECs. Media optimization studies with 2D cultures revealed that endothelial growth media was appropriate for the maintenance of both cell types. Suspension culture of hS/PCs in agarose microwells revealed that prolonged culture in EGM2 led to the upregulation of cellular expression of key stem/progenitor markers and differentiation markers. After encapsulation in a bioorthogonally constructed, cell‐adhesive, and cell‐permissive HA hydrogel and maintained in EGM2, the spheroids underwent dramatic morphological changes to produce multilobular microtissues with individual lobules interconnected with F‐actin‐rich structures and encased in a fibronectin cage. This was accompanied by upregulation of stem/progenitor and differentiation markers. Coculture of hS/PCs spheroids with HUVECs without direct contact between the two cell types led to further differentiation and maturation of the microtissues through endothelial cell secreted angiocrine signals. The biodegradability and biocompatibility of the cell‐free hydrogel were confirmed by hydrogel implantation in a rat model of parotid gland resection.

## AUTHOR CONTRIBUTIONS

**Mugdha Pol, Apoorva S. Metkari:** Investigation; methodology; writing—original draft and revision; formal analysis; data curation. **Stephen M. Frazier, Joshua B. Macugay, Hanyuan Gao:** Investigation; methodology. **Robert L. Witt:** Conceptualization; resources; writing—review and editing. **David M. Cognetti:** Resources; writing—review and editing. **Charles‐Antoine Assenmacher:** Resources, formal analysis; writing—review and editing. **Xinqiao Jia:** Conceptualization; funding acquisition; writing—review and editing; methodology; formal analysis; data curation; supervision.

## CONFLICT OF INTEREST STATEMENT

The authors declare no conflict of interest.

## Supporting information


**Figure S1.** Bright field images of hS/PCs cultured in 2D in different media conditions. Scale bar: 100 μm.
**Figure S2.** Representative confocal images of hS/PCs cultured in 2D in different media conditions. Nuclei are stained with Hoechst.
**Figure S3.** Bright‐field images of HUVECs cultured in 2D in different media conditions. Scale bar: 100 μm.
**Figure S4.** Representative confocal images of HUVECs cultured in 2D in different media conditions. Nuclei are stained with Hoechst.
**Figure S5.** hS/PC spheroids in suspension culture in different media conditions. Representative confocal images showing keratin 5 staining (green), are maintained in different media conditions on day 3 (A) and day 7 (B). F‐actin is stained with phalloidin 568 (red) and nuclei are stained with DAPI (blue). Scale bar: 20 μm.
**Figure S6.** Representative confocal images depicting NKCC1 acinar marker expression (green) in hS/PC spheroids cultured in suspension in different media conditions at day 3 (A) and day 7 (B). Nuclei were stained with DAPI (blue). F‐actin is stained with phalloidin 568 (red). Scale bar = 40 μm.
**Figure S7.** Brightfield images of HUVECs cultured on RGD‐TCO modified HA‐Tz gel as 2.5D monocultures on days 2, 7, and 14. Scale bar: 100 μm.
**Figure S8.** Three‐dimensional reconstructions of z‐stack confocal images of the coculture before (A) and after (B, C) mechanical separation. Epithelial spheroids, produced using hS/PCs stained with CellTrace™ Yellow, were embedded in the HA gel, and HUVECs stained with CellTrace™ Far Red were added on top of the hydrogel. After HUVECs were attached, the endothelial cell layer was dissected using a sharp scalpel. Images were acquired using an LSM 880 laser scanning confocal microscope. Z‐stack images were captured using a Fluar 5×/0.25 NA air objective, with a step size ranging from 10 to 30 μm, both prior to and following mechanical separation of the two layers. 3D reconstructions of the z‐stacks were generated with different *x*‐, *y*‐, and *z*‐dimensions to confirm physical separation of the two cell types before RNA extraction.
**Table S1.** Primer pairs used for qPCR.
**Table S2.** Antibodies used for immunostaining.

## Data Availability

The data that support the findings of this study are available from the corresponding author upon reasonable request.

## References

[1] Morales EA , Wang S . Salivary gland developmental mechanics. Curr Top Dev Biol. 2024;160:1‐30. doi:10.1016/bs.ctdb.2024.05.002 38937029

[2] Sasportas LS , Hosford DN , Sodini MA , et al. Cost‐effectiveness landscape analysis of treatments addressing xerostomia in patients receiving head and neck radiation therapy. Oral Surg Oral Med Oral Pathol Oral Radiol. 2013;116(1):e37‐e51. 23643579 10.1016/j.oooo.2013.02.017 PMC4018820

[3] Salum FG , Medella‐Junior FAC , Figueiredo MAZ , Cherubini K . Salivary hypofunction: an update on therapeutic strategies. Gerodontology. 2018;35(4):305‐316. doi:10.1111/ger.12353 29956369

[4] Chibly AM , Nguyen T , Limesand KH . Palliative care for salivary gland dysfunction highlights the need for regenerative therapies: a review on radiation and salivary gland stem cells. J Palliat Care Med. 2014;4(4):180‐186. doi:10.4172/2165-7386.1000180 PMC4675470 26693098

[5] Ozdemir T , Fowler EW , Hao Y , et al. Biomaterials‐based strategies for salivary gland tissue regeneration. Biomater Sci. 2016;4(4):592‐604. doi:10.1039/c5bm00358j 26878077 PMC4803517

[6] Pradhan‐Bhatt S , Harrington DA , Duncan RL , Jia X , Witt RL , Farach‐Carson MC . Implantable three‐dimensional salivary spheroid assemblies demonstrate fluid and protein secretory responses to neurotransmitters. Tissue Eng Part A. 2013;19(13–14):1610‐1620. doi:10.1089/ten.TEA.2012.0301 23442148 PMC3665323

[7] Ozdemir T , Fowler EW , Liu S , et al. Tuning hydrogel properties to promote the assembly of salivary gland spheroids in 3D. ACS Biomater Sci Eng. 2016;2(12):2217‐2230. doi:10.1021/acsbiomaterials.6b00419 27990487 PMC5155608

[8] Srinivasan PP , Patel VN , Liu S , et al. Primary salivary human stem/progenitor cells undergo microenvironment‐driven acinar‐like differentiation in hyaluronate hydrogel culture. Stem Cells Transl Med. 2017;6(1):110‐120. doi:10.5966/sctm.2016-0083 28170182 PMC5442728

[9] Fowler EW , Ravikrishnan A , Witt RL , Pradhan‐Bhatt S , Jia X . RGDSP‐decorated hyaluronate hydrogels facilitate rapid 3D expansion of amylase‐expressing salivary gland progenitor cells. ACS Biomater Sci Eng. 2021;7(12):5749‐5761. doi:10.1021/acsbiomaterials.1c00745 34781679 PMC8680203

[10] Fowler EW , van Venrooy EJ , Witt RL , Jia X . A TGFβR inhibitor represses keratin‐7 expression in 3D cultures of human salivary gland progenitor cells. Sci Rep. 2022;12(1):15008. doi:10.1038/s41598-022-19253-x 36056161 PMC9440137

[11] Metkari AS , Fowler EW , Witt RL , Jia X . Matrix degradability contributes to the development of salivary gland progenitor cells with secretory functions. ACS Appl Mater Interfaces. 2023;15(27):32148‐32161. doi:10.1021/acsami.3c03064 37364369 PMC10529452

[12] Fowler EW , Witt RL , Jia X . Basement membrane mimetic hydrogel cooperates with rho‐associated protein kinase inhibitor to promote the development of acini‐like salivary gland spheroids. Adv Nanobiomed Res. 2023;3(11):2300088. doi:10.1002/anbr.202300088 38645834 PMC11031203

[13] Metkari AS , Witt RL , Cognetti DM , Dhong C , Jia X . Promoting polarization and differentiation of primary human salivary gland stem/progenitor cells in protease‐degradable hydrogels via ROCK inhibition. ACS Appl Mater Interfaces. 2025;17(12):18083‐18095. doi:10.1021/acsami.4c22507 40095914 PMC12750517

[14] Ozdemir T , Srinivasan PP , Zakheim DR , et al. Bottom‐up assembly of salivary gland microtissues for assessing myoepithelial cell function. Biomaterials. 2017;142:124‐135. doi:10.1016/j.biomaterials.2017.07.022 28734180 PMC5561745

[15] Lin RZ , Chang HY . Recent advances in three‐dimensional multicellular spheroid culture for biomedical research. Biotechnol J. 2008;3(9–10):1172‐1184. doi:10.1002/biot.200700228 18566957

[16] Sakai T , Larsen M , Yamada KM . Fibronectin requirement in branching morphogenesis. Nature. 2003;423(6942):876‐881. doi:10.1038/nature01712 12815434

[17] Nelson CM . Geometric control of tissue morphogenesis. Biochim Biophys Acta. 2009;1793(5):903‐910. doi:10.1016/j.bbamcr.2008.12.014 19167433 PMC2683193

[18] Wang S , Sekiguchi R , Daley WP , Yamada KM . Patterned cell and matrix dynamics in branching morphogenesis. J Cell Biol. 2017;216(3):559‐570. doi:10.1083/jcb.201610048 28174204 PMC5350520

[19] Patel VN , Hoffman MP . Salivary gland development: a template for regeneration. Semin Cell Dev Biol. 2014;25‐26:52‐60. doi:10.1016/j.semcdb.2013.12.001 PMC3988231 24333774

[20] Kwon HR , Nelson DA , DeSantis KA , Morrissey JM , Larsen M . Endothelial cell regulation of salivary gland epithelial patterning. Development. 2017;144(2):211‐220. doi:10.1242/dev.142497 28096213 PMC5394760

[21] Schlieve CR , Mojica SG , Holoyda KA , Hou X , Fowler KL , Grikscheit TC . Vascular endothelial growth factor (VEGF) bioavailability regulates angiogenesis and intestinal stem and progenitor cell proliferation during postnatal small intestinal development. PLoS One. 2016;11(3):e0151396. doi:10.1371/journal.pone.0151396 26978773 PMC4792464

[22] Matsumoto K , Yoshitomi H , Rossant J , Zaret KS . Liver organogenesis promoted by endothelial cells prior to vascular function. Science. 2001;294(5542):559‐563. doi:10.1126/science.1063889 11577199

[23] Lammert E , Cleaver O , Melton D . Induction of pancreatic differentiation by signals from blood vessels. Science. 2001;294(5542):564‐567. doi:10.1126/science.1064344 11577200

[24] Lazarus A , Del‐Moral PM , Ilovich O , Mishani E , Warburton D , Keshet E . A perfusion‐independent role of blood vessels in determining branching stereotypy of lung airways. Development. 2011;138(11):2359‐2368. doi:10.1242/dev.060723 21558382 PMC3091498

[25] Rafii S , Butler JM , Ding BS . Angiocrine functions of organ‐specific endothelial cells. Nature. 2016;529(7586):316‐325. doi:10.1038/nature17040 26791722 PMC4878406

[26] Oria VO , Erler JT . Tumor angiocrine signaling: novel targeting opportunity in cancer. Cells. 2023;12(20):2510. doi:10.3390/cells12202510 37887354 PMC10605017

[27] Mendelson A , Frenette PS . Hematopoietic stem cell niche maintenance during homeostasis and regeneration. Nat Med. 2014;20(8):833‐846. doi:10.1038/nm.3647 25100529 PMC4459580

[28] Ingthorsson S , Sigurdsson V , Fridriksdottir A , et al. Endothelial cells stimulate growth of normal and cancerous breast epithelial cells in 3D culture. BMC Res Notes. 2010;3:184. doi:10.1186/1756-0500-3-184 20609224 PMC2909928

[29] Hagiwara M , Peng F , Ho CM . *In vitro* reconstruction of branched tubular structures from lung epithelial cells in high cell concentration gradient environment. Sci Rep. 2015;5:8054. doi:10.1038/srep08054 25623780 PMC4306969

[30] Jaramillo M , Mathew S , Mamiya H , Goh SK , Banerjee I . Endothelial cells mediate islet‐specific maturation of human embryonic stem cell‐derived pancreatic progenitor cells. Tissue Eng Part A. 2015;21(1–2):14‐25. doi:10.1089/ten.TEA.2014.0013 24943736 PMC4293092

[31] Kao DI , Lacko LA , Ding BS , et al. Endothelial cells control pancreatic cell fate at defined stages through EGFL7 signaling. Stem Cell Rep. 2015;4(2):181‐189. doi:10.1016/j.stemcr.2014.12.008 PMC4325230 25601205

[32] de Paula F , Teshima THN , Hsieh R , Souza MM , Nico MMS , Lourenco SV . Overview of human salivary glands: highlights of morphology and developing processes. Anat Rec (Hoboken). 2017;300(7):1180‐1188. doi:10.1002/ar.23569 28192873

[33] Nan W , He Y , Wang S , Zhang Y . Molecular mechanism of VE‐cadherin in regulating endothelial cell behaviour during angiogenesis. Front Physiol. 2023;14:1234104. doi:10.3389/fphys.2023.1234104 37601629 PMC10433914

[34] Privratsky JR , Newman PJ . PECAM‐1: regulator of endothelial junctional integrity. Cell Tissue Res. 2014;355(3):607‐619. doi:10.1007/s00441-013-1779-3 24435645 PMC3975704

[35] Pol M , Gao H , Zhang H , George OJ , Fox JM , Jia X . Dynamic modulation of matrix adhesiveness induces epithelial‐to‐mesenchymal transition in prostate cancer cells in 3D. Biomaterials. 2023;299:122180. doi:10.1016/j.biomaterials.2023.122180 37267701 PMC10330660

[36] Gao H , Pol M , Makara CA , et al. Bio‐orthogonal tuning of matrix properties during 3D cell culture to induce morphological and phenotypic changes. Nat Protoc. 2025;20:727‐778. doi:10.1038/s41596-024-01066-z 39501109 PMC11898115

[37] Cotrim AP , Sowers A , Mitchell JB , Baum BJ . Prevention of irradiation‐induced salivary hypofunction by microvessel protection in mouse salivary glands. Mol Ther. 2007;15(12):2101‐2106. doi:10.1038/sj.mt.6300296 17726456

[38] Andrée B , Ichanti H , Kalies S , et al. Formation of three‐dimensional tubular endothelial cell networks under defined serum‐free cell culture conditions in human collagen hydrogels. Sci Rep. 2019;9(1):5437. doi:10.1038/s41598-019-41985-6 30932006 PMC6443732

[39] Bautch VL . VEGF‐directed blood vessel patterning: from cells to organism. Cold Spring Harb Perspect Med. 2012;2(9):a006452. doi:10.1101/cshperspect.a006452 22951440 PMC3426816

[40] Xu X , Sabanayagam CR , Harrington DA , Farach‐Carson MC , Jia X . A hydrogel‐based tumor model for the evaluation of nanoparticle‐based cancer therapeutics. Biomaterials. 2014;35(10):3319‐3330. doi:10.1016/j.biomaterials.2013.12.080 24447463 PMC3929180

[41] Hsu JC , Yamada KM . Salivary gland branching morphogenesis—recent progress and future opportunities. Int J Oral Sci. 2010;2(3):117‐126. doi:10.4248/IJOS10042 21125789 PMC3168569

[42] Walker JL , Menko AS , Khalil S , et al. Diverse roles of E‐cadherin in the morphogenesis of the submandibular gland: insights into the formation of acinar and ductal structures. Dev Dyn. 2008;237(11):3128‐3141. doi:10.1002/dvdy.21717 18816447 PMC2771181

[43] Wang S , Matsumoto K , Lish SR , Cartagena‐Rivera AX , Yamada KM . Budding epithelial morphogenesis driven by cell‐matrix versus cell‐cell adhesion. Cell. 2021;184(14):3702‐3716. doi:10.1016/j.cell.2021.05.015 34133940 PMC8287763

[44] Feijão T , Neves MI , Sousa A , et al. Engineering injectable vascularized tissues from the bottom‐up: dynamics of in‐gel extra‐spheroid dermal tissue assembly. Biomaterials. 2021;279:121222. doi:10.1016/j.biomaterials.2021.121222 34736148

[45] Chlasta J , Milani P , Runel G , et al. Variations in basement membrane mechanics are linked to epithelial morphogenesis. Development. 2017;144(23):4350‐4362. doi:10.1242/dev.152652 29038305

[46] Yu Y , Huang H , Ye J , et al. 3D spheroids facilitate differentiation of human adipose‐derived mesenchymal stem cells into hepatocyte‐like cells via p300‐mediated H3K56 acetylation. Stem Cells Transl Med. 2024;13(2):151‐165. doi:10.1093/stcltm/szad076 37936499 PMC10872693

[47] Mélou C , Pellen‐Mussi P , Novello S , et al. Spheroid culture system, a promising method for chondrogenic differentiation of dental mesenchymal stem cells. Biomedicine. 2023;11:11. doi:10.3390/biomedicines11051314 PMC10216067 37238984

[48] Hosseini ZF , Nelson DA , Moskwa N , Sfakis LM , Castracane J , Larsen M . FGF2‐dependent mesenchyme and laminin‐111 are niche factors in salivary gland organoids. J Cell Sci. 2018;131(4):jcs208728. doi:10.1242/jcs.208728 29361536 PMC5868949

[49] Limesand KH , Avila JL , Victory K , et al. Insulin‐like growth factor‐1 preserves salivary gland function after fractionated radiation. Int J Radiat Oncol Biol Phys. 2010;78(2):579‐586. doi:10.1016/j.ijrobp.2010.03.035 20638195 PMC2939244

[50] Grundmann O , Fillinger JL , Victory KR , Burd R , Limesand KH . Restoration of radiation therapy‐induced salivary gland dysfunction in mice by post therapy IGF‐1 administration. BMC Cancer. 2010;10:417. doi:10.1186/1471-2407-10-417 20698985 PMC3087323

[51] Toan NK , Kim SA , Ahn SG . Ascorbic acid induces salivary gland function through TET2/acetylcholine receptor signaling in aging SAMP1/Klotho(−/−) mice. Aging (Albany NY). 2022;14(15):6028‐6046. doi:10.18632/aging.204213 35951355 PMC9417236

[52] Moskwa N , Mahmood A , Nelson DA , Altrieth AL , Forni PE , Larsen M . Single‐cell RNA sequencing reveals PDGFRα+ stromal cell subpopulations that promote proacinar cell differentiation in embryonic salivary gland organoids. Development. 2022;149(6):dev200167. doi:10.1242/dev.200167 35224622 PMC8977102

[53] Tunica DG , Yin X , Sidibe A , et al. Proteomic analysis of the secretome of human umbilical vein endothelial cells using a combination of free‐flow electrophoresis and nanoflow LC‐MS/MS. Proteomics. 2009;9(21):4991‐4996. doi:10.1002/pmic.200900065 19810032

[54] Patterson J , Hubbell JA . Enhanced proteolytic degradation of molecularly engineered PEG hydrogels in response to MMP‐1 and MMP‐2. Biomaterials. 2010;31(30):7836‐7845. doi:10.1016/j.biomaterials.2010.06.061 20667588

[55] O'Keefe KJ , DeSantis KA , Altrieth AL , et al. Regional differences following partial salivary gland resection. J Dent Res. 2020;99(1):79‐88. doi:10.1177/0022034519889026 31765574 PMC6927217

[56] Vegas AJ , Veiseh O , Doloff JC , et al. Combinatorial hydrogel library enables identification of materials that mitigate the foreign body response in primates. Nat Biotechnol. 2016;34(3):345‐352. doi:10.1038/nbt.3462 26807527 PMC4904301

[57] Murray LA , Habiel DM , Hohmann M , et al. Antifibrotic role of vascular endothelial growth factor in pulmonary fibrosis. JCI Insight. 2017;2(16):e92192. doi:10.1172/jci.insight.92192 28814671 PMC5621899

