# Supplementary material for: Fostering cell–cell interactions and integrating angiocrine factors to promote the development of salivary microtissues in 3D
Source: Bioeng Transl Med. 2026 Feb 18;11(3):e70118. doi: 10.1002/btm2.70118 (PMC13247410; doi:10.1002/btm2.70118)
Supplement: Supplementary file 1 — Figure S1. Bright field images of hS/PCs cultured in 2D in different media conditions. Scale bar: 100 μm. Figure S2. Representative confocal images of hS/PCs cultured in 2D in different media conditions. Nuclei are stained with Hoechst. Figure S3. Bright‐field images of HUVECs cultured in 2D in different media conditions. Scale bar: 100 μm. Figure S4. Representative confocal images of HUVECs cultured in 2D in different media conditions. Nuclei are stained with Hoechst. Figure S5. hS/PC spheroids in suspension culture in different media conditions. Representative confocal images showing keratin 5 staining (green), are maintained in different media conditions on day 3 (A) and day 7 (B). F‐actin is stained with phalloidin 568 (red) and nuclei are stained with DAPI (blue). Scale bar: 20 μm. Figure S6. Representative confocal images depicting NKCC1 acinar marker expression (green) in hS/PC spheroids cultured in suspension in different media conditions at day 3 (A) and day 7 (B). Nuclei were stained with DAPI (blue). F‐actin is stained with phalloidin 568 (red). Scale bar = 40 μm. Figure S7. Brightfield images of HUVECs cultured on RGD‐TCO modified HA‐Tz gel as 2.5D monocultures on days 2, 7, and 14. Scale bar: 100 μm. Figure S8. Three‐dimensional reconstructions of z‐stack confocal images of the coculture before (A) and after (B, C) mechanical separation. Epithelial spheroids, produced using hS/PCs stained with CellTrace™ Yellow, were embedded in the HA gel, and HUVECs stained with CellTrace™ Far Red were added on top of the hydrogel. After HUVECs were attached, the endothelial cell layer was dissected using a sharp scalpel. Images were acquired using an LSM 880 laser scanning confocal microscope. Z‐stack images were captured using a Fluar 5×/0.25 NA air objective, with a step size ranging from 10 to 30 μm, both prior to and following mechanical separation of the two layers. 3D reconstructions of the z‐stacks were generated with different x‐, y‐, and z‐dimensions to conf [file BTM2-11-e70118-s001.docx]

**SUPPLEMENTARY INFORMATION**

**Experimental Methods**

***Cell maintenance.***

With institutional approval and following our reported procedures ^6, 8, 9, 11, 33^ hS/PCs were isolated from parotid tissues obtained from consented patients undergoing parotidectomy at Christiana Care (Newark, DE) and Thomas Jefferson University Hospital (Philadelphia, PA). Isolated hS/PCs were maintained and passaged in Corning^TM^ HepatoSTIM media (HEP, Corning, NY) supplemented with epidermal growth factor (EGF), penicillin and streptomycin, and amphotericin B as previously described.^8, 9, 11^ HUVECs, purchased from ATCC (Manassas, VA), were plated on 0.1% gelatin-coated tissue culture plates and maintained in endothelial growth media 2 (EGM-2, Lonza, Basel) per vendor’s instructions. At 80% confluency, cells were detached with 0.05% Trypsin EDTA (3 min), washed with PBS, centrifuged at 400 *g* (5 min), and split for subculture. Experiments were conducted using hS/PCs at passages between 4-8 and HUVECs at passages between 2-6. To quantify cell proliferation, cells were stained with Hoechst (1:1000) and imaged using a Zeiss LSM 880 confocal microscope (Carl Zeiss, Oberkochen, Germany). The total number of cells was calculated by multiplying the number of cells in the field of view in a confocal image, enumerated using ImageJ, by the total area of the well. The data was normalized to the cell seeding density on day 0. Three biological replicates were performed and 10 images from each replicate were analyzed.

***Formation of hS/PC spheroids.***

Agarose microwells were prepared using a plastic micro-mold (MicroTissues Inc., Sharon, MA) with 256 circular recesses (300 μm wide, 800 μm deep, arranged in a 16 × 16 array), following our reported procedure.^14^ Prior to cell seeding, the agarose microwells were transferred into a 12-well cell culture plate and equilibrated in the desired cell culture media for 2 h at 37°C. hS/PCs were suspended in media supplemented with 0.6 mg/mL reduced growth factor basement membrane extract (GFR-BME, R&D Systems, Minneapolis, MN) at 0.3×10^6^ cells/mL. The cell suspension (150 μL) was aliquoted into each microwell. After the cells settled down at the bottom of the well, the media on top of the microwells was carefully removed and replaced with 140 μL of fresh media containing 0.6 mg/mL basement membrane extract. Additional media without basement membrane extract (300 μL) was added around the agarose replica in the cell culture plate. Cells were maintained in suspension for 7 days without media change. Brightfield images of self-assembled spheroids were captured using an Eclipse Ti-E microscope (Nikon, Tokyo, Japan). Separately, spheroids in microwells were incubated with a Live/dead staining cocktail (Thermo Fisher Scientific) of calcein AM (1:500), ethidium homodimer (1:1000), and Hoechst 33342 (1:100) for 20 min at 37°C on days 3 and 7. After removing the dye solution, the wells were inspected using an LSM 880 confocal microscope (Carl Zeiss, Germany) with a Fluar 5×/0.25 air objective, and images were captured as 9.6 μm z-stacks and processed as maximum intensity projections. Cell viability was calculated from 10 random images for each condition using ImageJ (National Institutes of Health, Bethesda, MD) as previously described.^34, 35^

***Development of salivary gland microtissues.***

The hydrogel building blocks, including tetrazine-modified HA (HA-Tz), *trans*-cyclooctene (TCO)-functionalized cell adhesive peptide (RGD-TCO), and norbornene-functionalized, protease-degradable crosslinker (SMR-bisNb, VPMS↓MRGG), were prepared following our previously reported methods. ^34-36^ To prepare epithelial cell-laden hydrogels, hS/PC spheroids were gently aspirated from the agarose microwell, centrifuged at 500 rpm for 2 min, and dispersed in a fresh EGM2 solution containing dissolved HA-Tz and SMR-bisNb at a Tz/Nb molar ratio of 2/1. The resultant epithelial construct, containing 500-600 hS/PC spheroids/mL, was incubated at 37°C for 1 h before EGM2 media was added. After incubation at 37°C for 4 h on day 0, the medium was removed and fresh EGM2 containing RGD-TCO (4 mM) was added to the constructs. After incubation at 37 °C for 24 h, the RGD-TCO reservoir was removed, ~1500 HUVECs were seeded on top of the hS/PC-laden construct, and the coculture was maintained in the EGM2 for 14 days, with media refreshments every three days. Epithelial monocultures were established by culturing pre-assembled hS/PCs spheroids in HA gels alone, and endothelial monocultures were produced by seeding HUVECs on top of the cell-free HA gels 24 h after interfacial RGD tagging. For transcript analysis, constructs (300 µL) were developed in a 12 mm PTFE cell culture insert (Millicell 0.4 μm, EMD Millipore Corporation, Burlington, MA). For immunofluorescence, smaller constructs (50 µL) were produced in a 48-well glass-bottom MatTek plate (MatTek Corporation, Ashland, MA).

***Quantitative polymerase chain reaction (qPCR).***

After the 3D coculture was terminated, the constructs were washed with PBS, and the endothelial monolayer was carefully dissected with a sharp scalpel; successful separation of the two cell types was confirmed by bright-field and confocal microscopy. The endothelial layer and the underlying epithelial constructs were collected separately and snap-frozen using a dry ice/isopropanol mixture. For monoculture controls, cells, spheroids, or cell-laden hydrogels were directly snap-frozen. The frozen samples were crushed vigorously with a pestle to form a gel slurry. Next, Trizol (Invitrogen, Carlsbad, CA) was added to lyse the cells. mRNA extraction was performed following our reported method.^11, 13, 34, 35^ The extracted RNA was reconstituted in 20 μL nuclease-free water. RNA quantification and purity were assessed using a NanoDrop 2000 spectrophotometer (Nanodrop Technologies, LLC, Wilmington, DE). Using QuantiTect Reverse Transcription Kit (Qiagen, Germantown, MD), a total of 0.5 µg of RNA was reverse transcribed to cDNA following the manufacturer’s protocol. An Applied Biosystems 7300 real-time PCR machine was used to perform and monitor sequence-specific amplification. The PCR reaction mixture was prepared by combining Power SYBR™ green PCR master mix (Applied Biosystems, Foster City, CA), cDNA, and target-specific primers to a total volume of 20 μL per reaction. All primers were purchased from Integrated DNA Technologies (Coralville, IA), and the complete primer sequences are available in Table S1. Glyceraldehyde 3-phosphate dehydrogenase *(GAPDH)* was used as a reference gene. The obtained C_T_ values were normalized to *GAPDH*. The fold changes were calculated using the ΔΔC_T_ method. Experiments were conducted using three biological replicates, with three technical replicates measured in duplicate.

***Immunofluorescence.***

Hydrogel-derived cellular constructs were fixed with 4% paraformaldehyde (PFA), permeabilized in 0.2% Triton, blocked in 3% bovine serum albumin (BSA) in 1X phosphate buffered saline (PBS), and incubated with the primary antibody at an appropriate dilution in 3% BSA. Antibody information and staining conditions can be found in Table S2. After the primary antibody solution was removed, samples were washed thrice with 1X PBS and incubated with the secondary (Alexa Fluor^TM^ 488 goat anti-mouse/rabbit IgG) solution (1:250 in 3% BSA) containing Alexa Fluor^TM^ 568 phalloidin for 4 h at room temperature. The antibody solution was aspirated, and the sample was washed three times with Tween 20-containing 1X PBS (PBST), followed by three washes with PBS. After cell nuclei were counterstained with DAPI at 1:1000 dilution in PBS for 1 h, samples were incubated in PBS for 24 h. Agarose microwell-derived hS/PC spheroids were encapsulated in RGD-free HA gels using HA-Tz and SMR-bisNb prior to fixation and staining. The stained constructs were inspected with an LSM 880 microscope with an Airyscan detector in Fast Airy mode. Using LD LCI Plan-Apochromat 25×/0.8 Imm Korr water/oil objective, images were captured as 30-70 μm z-stacks with 2 μm z-axis step size or as single slice. After Airyscan processing, z-stack images were saved as maximum intensity projections, and brightness was evenly adjusted using ZEN 3.0 SR software.

***Hydrogel implantation.***

Animal studies were performed as approved by the IACUC at the University of Delaware using 3-month-old Long-Evans rats (Boston, MA). Rats were anesthetized in a closed chamber purged with 3% isoflurane in oxygen at 1.0 L/min. The sedated animal was transferred to a surgical bench, and the oxygen/isoflurane mixture continued to be supplied through a nose cone at 1.0 L/min during the procedure. To prepare for surgery, the area near the cheek was shaved and cleaned with isopropanol and betadine wipes, and saline (3 mL) and buprenorphine (0.015 mg/mL) were administered subcutaneously and intraperitoneally, respectively. To locate the parotid gland, a small incision (~ 1 cm long, 3-5 mm deep) was made next to the right ear, and 1/3 of the gland was resected using surgical scissors. A cell-free HA gel (50 µL) was placed at the resection site, and the incision was sutured using 4.0 coated Vicryl braided sutures (Ethicon, Raritan, NJ). Hydrogel implantation was performed in three animals. The left parotid gland was used as the untreated control, and the resected gland without hydrogel treatment was also included. Animals recovered and resumed activity post-surgery.

***Intravital imaging.***

Fluorescently tagged HA hydrogels were prepared by adding Cy7-TCO (5 µM, Ruixi Biotech Co. Ltd., Shaanxi, China) to the gelling liquid. The Cy7-labeled gel was implanted in the resected gland of 3 rats, and gel degradation was monitored using IVIS Lumina III in vivo imaging system on days 0, 7,14, and 21. For background correction, 3 rats receiving Cy7-free HA gels were imaged under the same conditions. Animals were euthanized after 21 days, and the surgery site was inspected for the presence of any gel fragments.

***Histology.***

Seven and 21 days after implantation, rats were euthanized using CO_2,_ and the parotid gland was harvested and then fixed in 70% ethanol. The tissue samples were placed in cassettes and sequentially exposed to graded alcohols (Deacon Labs Inc., King of Prussia, PA), xylene (Fisher Chemical, Fair Lawn, NJ), and paraffin using a Pegasus processor (Leica, Buffalo Grove, IL). Five-micron tissue sections were cut using a Leica RM2255 microtome (Leica, Buffalo Grove, IL) and collected on Superfrost® Plus slides (Thermo Fisher Scientific, Fremont, CA). The slides were heated at 60°C for 1 h and stored at ambient temperature. Before staining, the slides were dewaxed using two changes of xylene, followed by one change of each of the graded alcohols (100%, 95%, and 80%). For H&E staining, the slides were immersed in Harris Hematoxylin (Stat Lab, McKinney, TX), 1% acid alcohol (Fisher Chemical, Fair Lawn, NJ), and ammonia water (Rowley Biochemicals, Danvers, MA) with one rinse in tap water after each, and finally EosinY/Phloxine B solution (Fisher Chemical, Fair Lawn, NJ). For Mason’s trichrome staining, the slides were placed into Bouin’s Fixative (Poly Scientific R&D Corp., Bay Shore, NY) in a 56˚C oven for 1 h, then cooled and washed in running tap water, followed by a rinse in distilled water. Slides were sequentially placed in Weigert's Iron Hematoxylin working solution and Biebrich Scarlet Acid Fuchsin, with a water rinse in between. The slides were placed in phosphotungstic/phosphomolybdic acid, followed by direct addition to the aniline blue solution. They were then rinsed in distilled water and placed in acetic acid. After dehydration in graded ethanol and xylene, the slides were then cover-slipped on the Sakura Tissue-Tek® Glas™ Automated coverslipper using Tissue-Tek mounting medium (Sakura Finetek USA Inc., Torrance, CA). The stained slides were imaged using the Carl Zeiss Axio Plan microscope (Carl Zeiss). Slides were evaluated by a pathologist unaware of the sample identification at the Penn Vet Comparative Pathology Core (CPC) Facility at the University of Pennsylvania (Philadelphia, PA).

***Statistics.***

Data from at least three biological repeats were collected across different cell passages. The student’s t-test with p < 0.05 was considered significant for comparison between the two groups. For comparison between multiple groups, one-way ANOVA followed by Post Hoc Tukey analysis, with p < 0.05 was considered significant. Statistical analyses were made using GraphPad Prism (San Diego, CA) version 9.2.0 or JMP Pro 15 (SAS Institute Inc.). Graphs were plotted using GraphPad Prism. Error bars represent the standard error of the mean, unless stated otherwise.

**
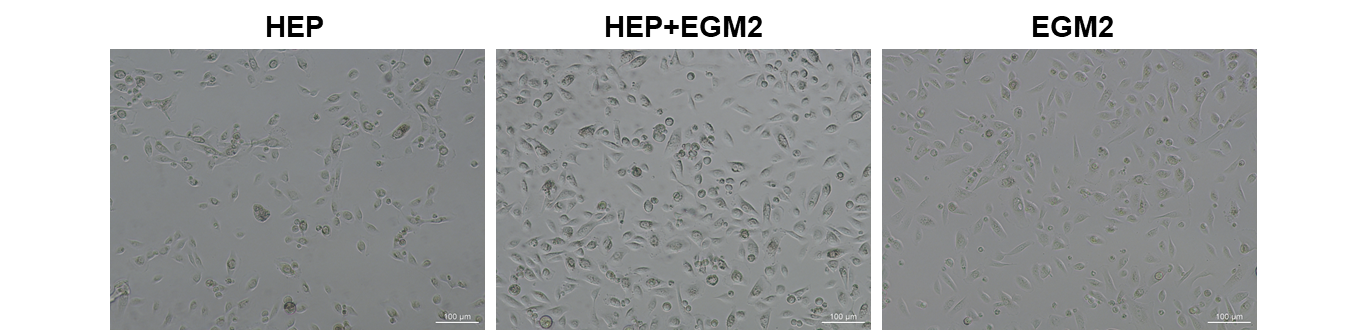
**

**Fig. S1**: Bright field images of hS/PCs cultured in 2D in different media conditions. Scale bar: 100 µm.


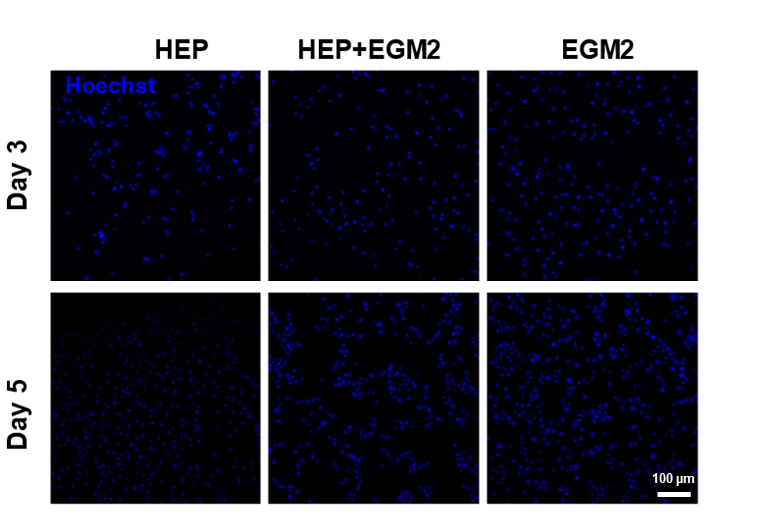


**Fig. S2**: Representative confocal images of hS/PCs cultured in 2D in different media conditions. Nuclei are stained with Hoechst.


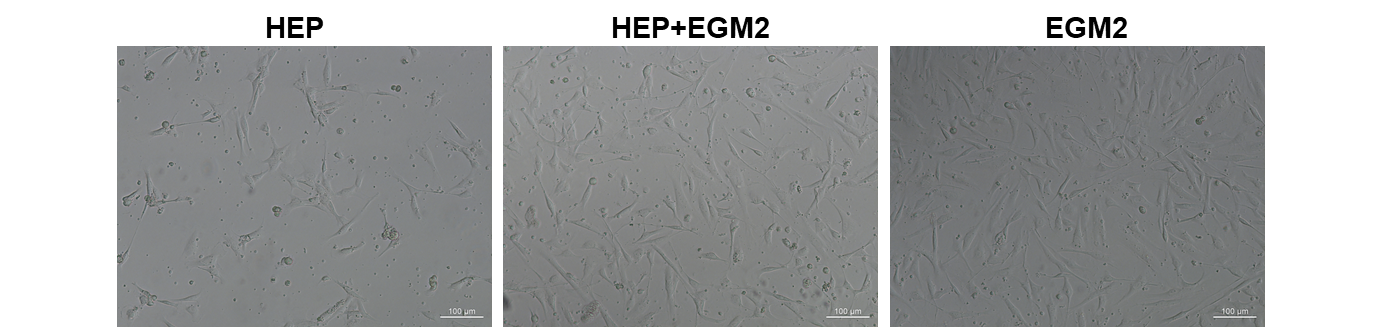


**Fig. S3**: Bright-field images of HUVECs cultured in 2D in different media conditions. Scale bar: 100 µm.


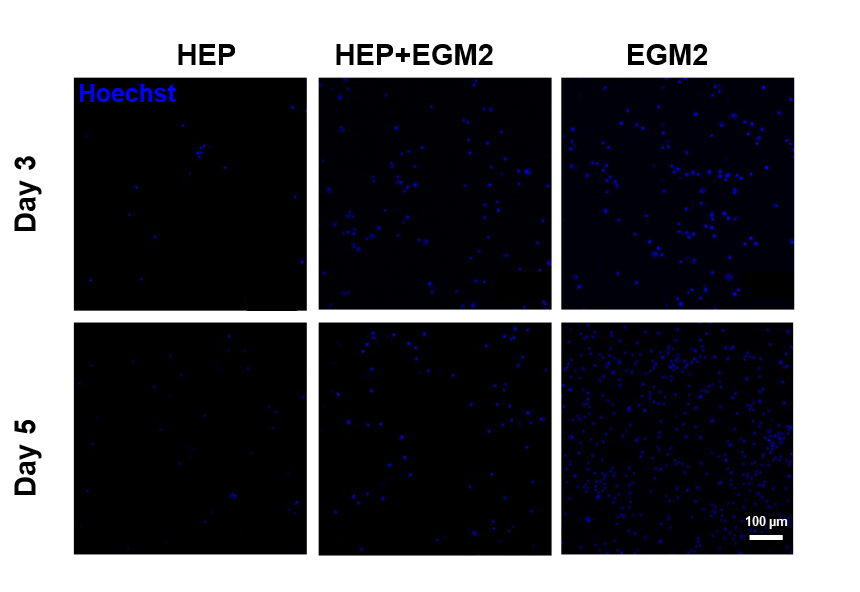


**Fig. S4**: Representative confocal images of HUVECs cultured in 2D in different media conditions. Nuclei are stained with Hoechst.


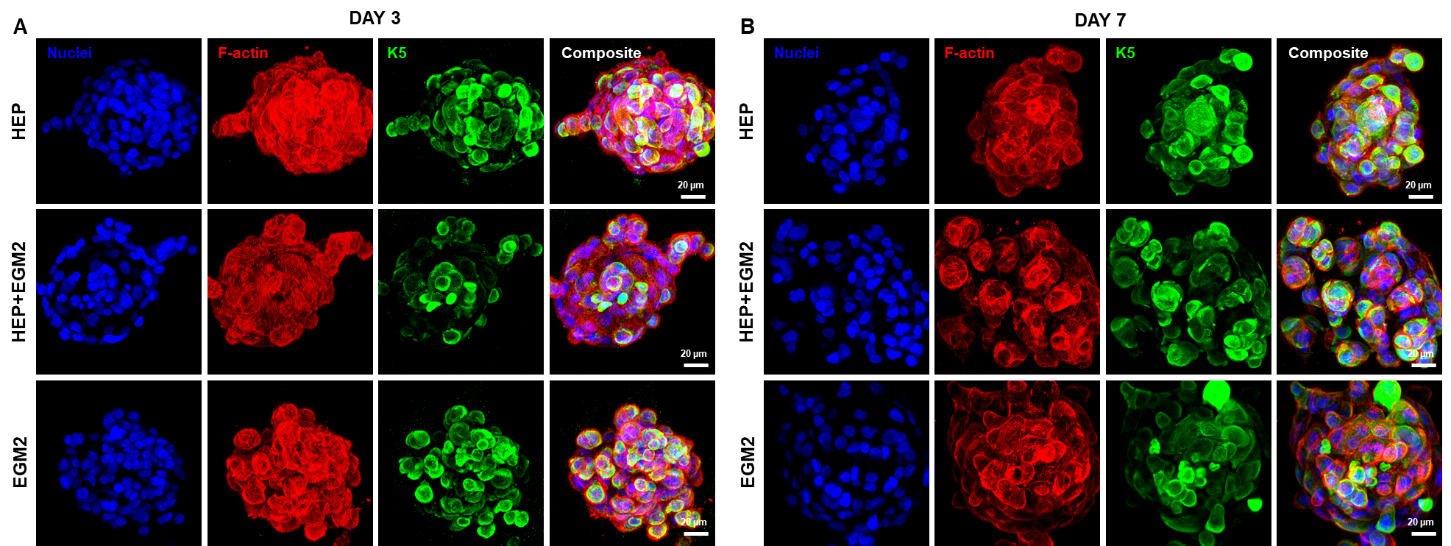


**Fig. S5**: hS/PC spheroids in suspension culture in different media conditions. Representative confocal images showing keratin 5 staining (green), are maintained in different media conditions on day 3 **(A)** and day 7 **(B)**. F-actin is stained with phalloidin 568 (red) and nuclei are stained with DAPI (blue). Scale bar: 20 µm.

**
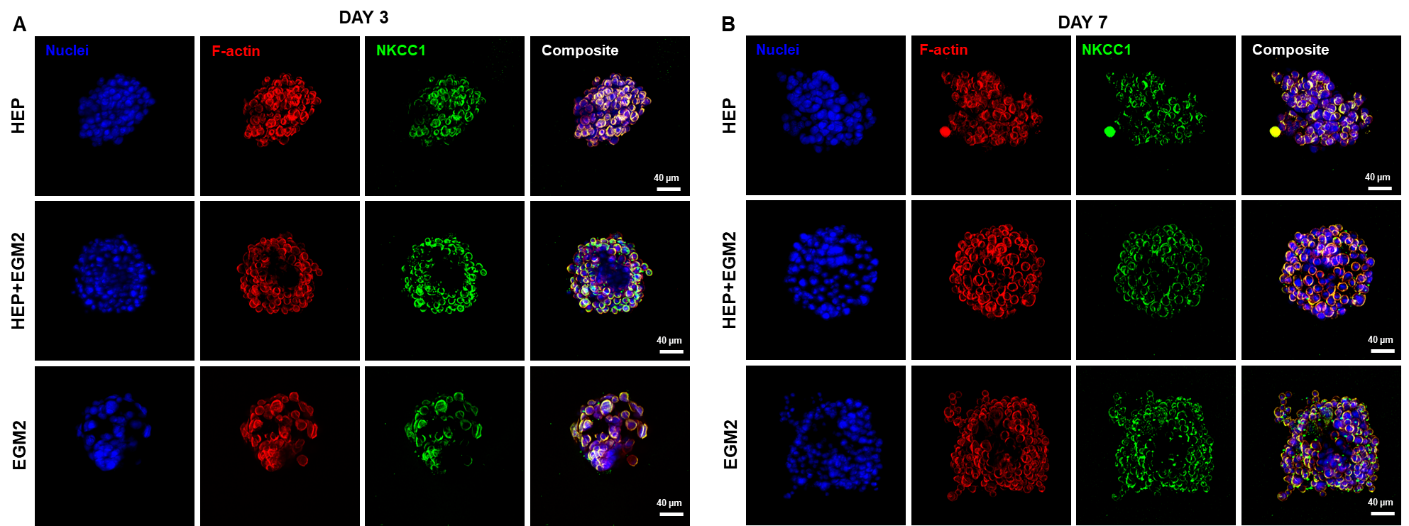
**

**Fig. S6**: Representative confocal images depicting NKCC1 acinar marker expression (green) in hS/PC spheroids cultured in suspension in different media conditions at day 3 **(A)** and day 7 **(B)**. Nuclei were stained with DAPI (blue). F-actin is stained with phalloidin 568 (red). Scale bar= 40 µm.


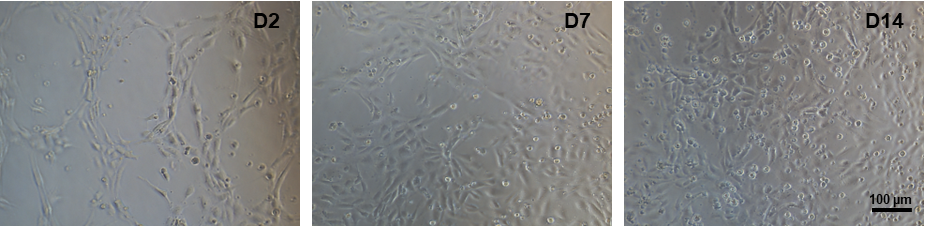


**Fig. S7**: Brightfield images of HUVECs cultured on RGD-TCO modified HA-Tz gel as 2.5D monocultures on days 2, 7, and 14. Scale bar: 100 µm.


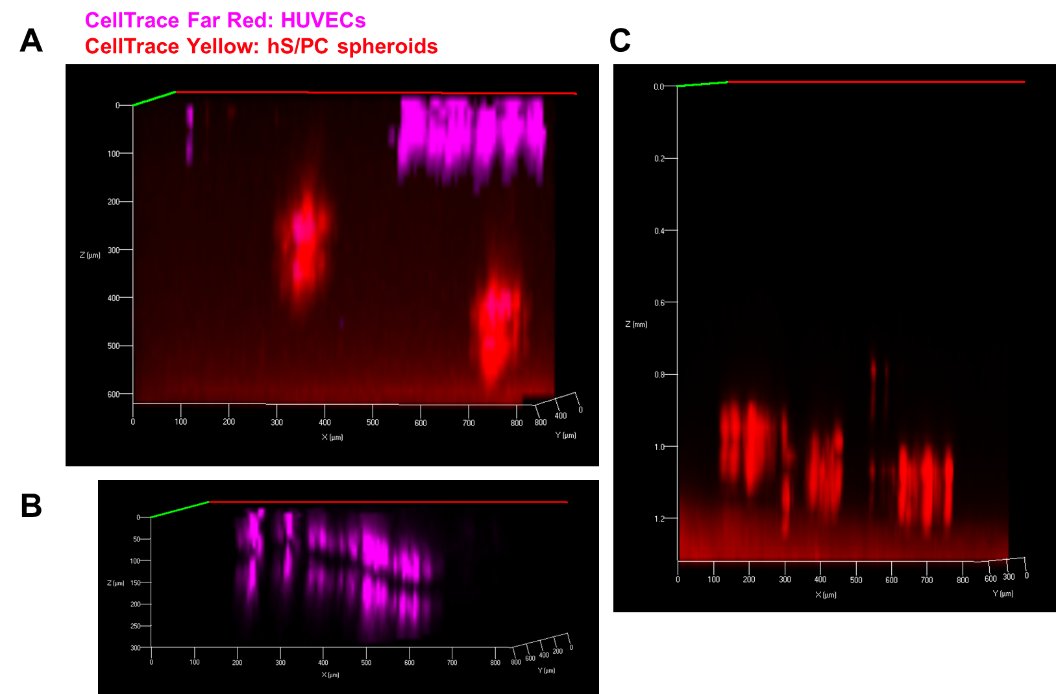


**Fig. S8**: Three-dimensional reconstructions of z-stack confocal images of the coculture before (A) and after (B, C) mechanical separation. Epithelial spheroids, produced using hS/PCs stained with CellTrace™ Yellow, were embedded in the HA gel, and HUVECs stained with CellTrace™ Far Red were added on top of the hydrogel. After HUVECs were attached, the endothelial cell layer was dissected using a sharp scalpel. Images were acquired using an LSM 880 laser scanning confocal microscope. Z-stack images were captured using a Fluar 5×/0.25 NA air objective, with a step size ranging from 10-30 µm, both prior to and following mechanical separation of the two layers. 3D reconstructions of the z-stacks were generated with different x-, y-, and z-dimensions to confirm physical separation of the two cell types before RNA extraction.

**Table S1:** Primer pairs used for qPCR.

| **Gene** | **Forward Primer** | **Reverse Primer** |
| --- | --- | --- |
| *GAPDH* | CAGCCTCAAGATCATCAGCA | TGTGGTCATGAGTCCTTCCA |
| *KRT5* | CGTGCCGCAGTTCTATATTCT | ACTTTGGGTTCTCGTGTCAG |
| *KRT14* | CACAGATCCCACTGGAAGAT | GATAATGAAGCTGTATTGATTGCC |
| *MYC* | CGGAACTCTTGTGCGTAAGG | TCATAGGTGATTGCTCAGGACAT |
| *KIT* | GTCTCCACCATCCATCCA | TCCATTCATTCTGCTTATTCTCA |
| *AMY* | CTCGGCACAGTTATTCGCAAGTGG | ACAGCCTAGCATCCCAGAAGGT |
| *AQP5* | CTGTCCATTGGCCTGTCTGTC | GGCTCATACGTGCCTTTGATC |
| *AQP3* | ACCAGCTTTTTGTTTCGGGC | GCCGGTCCTGGTCAA |
| *SLC12A2* | GGCAAGACTGCAACTCAACC | CACCTTTTCGTGCAACTGGG |
| *CDH1* | CGA GAG CTA CAC GTT CAC GG | GGG TGT CGA GGG AAA AAT AGG |
| *FN1* | ACCTACGGATGACTCGTGCTTTGA | CAAAGCCTAAGCACTGGCACAACA |
| *KRT7* | AAGAACCAGCGTGCCAAGT | TCCAGCTCCTCCTGCTTG |
| *KRT19* | CTGCCTCCAAGGTCCTCT | CCCATCCCTCTACCCAGAAG |
| *TFCP2L1* | GCCGCCTGCTTCCTGTTC | CTGCCCACCACTGCTCAAAG |
| *LAMA1* | GTGATGGCAACAGCGCAAA | GACCCAGTGATATTCTCTCCCA |
| *ETV4* | CAGACGGACTTCGCCTAC | GGAATGGTCGCAGAGGTT |
| *ETV5* | GTGATACTGACAGGTAAGCAACA | AGAGCAGGCAGCAAGGTA |
| *CD31* | GCTGAGTCTCACAAAGATCTAGGA | CCGTTCTAGAGTATCTGCTTTCCA |
| *CDH5* | GTCCTGCAGATCTCCGCAAT | TGTTGGCCGTGTTATCGTGA |
| *vWF* | CAACACCTGCATTTGCCGAA | TGACCTGTGACAAGGCACTC |
| *VEGFA* | TCACCAAGGCCAGCACATAG | TCGGCTTGTCACATTTTTCTTGTC |
| *FLT1* | CAGAAGGGCTCTGTGGAAAGT | GAGGTTCCTTGAACAGTGAGGTAT |

**Table S2**: Antibodies used for immunostaining

| **Target** | **Primary Antibody/**  **Catalog number** | **Permeabilization/Blocking** | **Dilution/Staining** |
| --- | --- | --- | --- |
| Keratin-14 | Abcam  ab2413 | 0.2% Triton, 2 h, r.t.;  3% BSA in PBS, 2 h, r.t. | 1:100 in 3% BSA  4 h, r.t. |
| Keratin-5 | Bio Legend  905501 | 0.2% Triton, 2 h, r.t.;  3% BSA in PBS, 2 h, r.t. | 1:100 in 3% BSA  4 h, r.t. |
| α- Amylase | Millipore Sigma  A8273 | 0.2% Triton, 2 h, r.t.;  3% BSA in PBS, 2 h, r.t. | 1:50 in 3% BSA  Overnight, 4°C, |
| Integrin β1 | Santa Cruz  sc-53711 | 0.2% Triton, 10 min, r.t.;  3% BSA in PBS, overnight, 4 °C | 1:100 in 3% BSA  Overnight, 4 °C, |
| CD44 | Invitrogen eBiosciences  14-0441-82 | 0.2% Triton, 10 min, r.t.;  3% BSA in PBS, overnight, 4 °C | 1:100 in 3% BSA  Overnight, 4 °C, |
| Fibronectin | Santa Cruz  sc-59826 | 0.2% Triton, 1 h, r.t.;  3% BSA in PBS, overnight, 4 °C | 1:100 in 3% BSA  Overnight, 4 °C |
| vWF | Abcam  ab154193 | 0.2% Triton, 2 h, r.t.;  3% BSA in PBS, overnight, 4 °C | 1:70 in 3% BSA  Overnight, 4 °C |
| CD31 | Abcam  ab28364 | 0.2% Triton, 10 min, r.t.;  3% BSA in PBS, overnight, 4 °C | 1:200 in 3% BSA  Overnight, 4 °C |
| VE-cadherin | Abcam  ab33168 | 0.2% Triton, 10 min, r.t.;  3% BSA in PBS, overnight, 4 °C | 1:100 in 3% BSA  Overnight, 4 °C |
| Vimentin | Genway Biotech  GWB-BBB094 | 0.2% Triton, 45 min, r.t;  3% BSA in PBS, 2 h, r.t. | 1:200 in 3% BSA  4 h, r.t. |
| NKCC1 | Cell Signaling  D13A9 | 0.2% Triton, 4 h, r.t.;  3% BSA in PBS, overnight, r.t. | 1:100 in 3% BSA  16 h, r.t. |
